# Facilitators and barriers to implementing AI-assisted diagnostic systems in primary healthcare services in low-resource settings: a mixed-methods study protocol

**DOI:** 10.3389/fdgth.2026.1967645

**Published:** 2026-09-29

**Authors:** Junhan Mu, Can Li, Ting Kang, Chengcheng Dong, Cong Chen, Bijie Ou, Huijuan Liang, Nan Zhang

**Affiliations:** Department of Health Management, School of Health Management, Inner Mongolia Medical University, Hohhot, China

**Keywords:** AI-assisted diagnostic systems, implementation science, low-resource settings, mixed-methods study, primary healthcare, technology acceptance

## Abstract

**Introduction:**

Although AI-assisted diagnostic systems are increasingly deployed in primary care globally, evidence from multiple countries indicates that deployment does not necessarily ensure clinical uptake. Resource-constrained and dispersed regions face implementation challenges, yet the mechanisms remain unclear. This mixed-methods protocol employs the Consolidated Framework for Implementation Research (CFIR) to systematically identify barriers and facilitators to AI implementation in primary care in such settings.

**Methods and analysis:**

This study adopts a sequential mixed-methods design. Phase I involves semi-structured in-depth interviews with multi-level stakeholders (defined by the 7P principle: Patients, Providers, Purchasers, Payers, Public, Policymakers, and Product suppliers), including primary care clinicians, facility managers, external administrators, and vendor staff. Purposive and maximum-variation sampling will cover diverse geographic areas and usage levels (high, low, and non-use). The interview guide will be developed based on the CFIR 2.0 domains. Interviews will be audio-recorded, transcribed verbatim, thematically analysed, and mapped to CFIR 2.0 constructs. Phase I will recruit professional stakeholders and patients until information saturation is reached. Phase II comprises a cross-sectional survey of primary care patients targeting a minimum of 482 valid responses, to identify and quantify patient-level acceptance of AI-assisted diagnosis and its influencing factors, informed by patients’ perspectives identified in Phase I. Phase III integrates findings from the first two phases and applies the Behaviour Change Wheel (BCW) and Implementation Mapping to formulate context-sensitive optimisation strategies.

**Ethics and dissemination:**

This study has been approved by the Medical Ethics Committee of Inner Mongolia Medical University (approval number: YKD202602099). All participants will provide written informed consent prior to data collection. Findings will be disseminated through peer-reviewed journal publications, national and international conference presentations, and policy briefs submitted to local health authorities. Beyond the pastoral regions of China, this protocol aims to generate an evidence base and a replicable methodological template (integrating CFIR 2.0, COM-B, and Implementation Mapping) that may inform the scaled implementation of AI in comparable resource-constrained and geographically dispersed health systems. The transferability of the findings to settings such as those in Central Asia, sub-Saharan Africa, and Small Island Developing States will be explicitly assessed as a research question rather than assumed.

## Highlights

This study is the first to systematically apply the CFIR framework to the implementation of AI-assisted diagnostic systems in primary care in low-resource, geographically dispersed regions of China, enabling multi-level, theoretically grounded identification of influencing factors and enriching implementation science applications in low-resource settings.

The study incorporates multi-level stakeholders (from frontline clinicians to external policymakers and technology vendors) defined using the 7P principle, providing a cross-level (individual, organisational, external environment, and institutional) comprehensive perspective that overcomes the single-perspective limitation of previous studies.

Through purposive sampling combined with maximum variation strategy, the study includes users with different usage levels (high-frequency, low-frequency, non-use), avoiding the bias of recruiting only AI proponents and thus obtaining more balanced and authentic views.

The study covers representative geographic types (agricultural, pastoral, and agro-pastoral areas) in low-resource regions, with findings potentially informing other low-resource and geographically dispersed settings.

The study employs a sequential mixed-methods design: the qualitative phase reveals mechanisms in depth, the quantitative phase quantifies patient-level relationships and tests the pathways underlying patient acceptance, and the third phase uses BCW and Implementation Mapping to translate findings into actionable implementation strategy recommendations, achieving a complete research chain from “mechanism understanding” to “strategy formulation” with strong practice-translation value.

## Limitations

Although this study is based on data from a specific region in China, its structural characteristics (geographical dispersion, low provider density, and weak digital infrastructure) show some similarities to those of resource-limited settings worldwide, while important differences in financing, governance, and institutional capacity must be acknowledged. We therefore argue for the transferability of theoretical mechanisms rather than statistical universality, and we treat transferability as a research question to be assessed rather than an expected outcome. To support such assessments, we provide a “context–structure” mapping framework that links the structural characteristics of the study sites (geographical dispersion, provider density, workforce composition, digital infrastructure, financing arrangements, and regulatory environment) to the dimensions along which transferability to other settings would need to be evaluated. To address social desirability and recall biases, we combine system logs (where available), field observation records, and a focus on recent specific events rather than general impressions. Despite these limitations, the methodological rigor (integrating CFIR 2.0, quantitative path modeling, and the BCW framework) offers a robust evidence base for AI policy development in analogous contexts.

## Introduction

### Global health equity and the strategic priority of primary health care

Primary Health Care (PHC) is the cornerstone of achieving Universal Health Coverage (UHC). The World Health Organization explicitly states that the core of UHC lies in ensuring that everyone has access to accessible, affordable, safe, effective, and people-centred health services (1). United Nations Sustainable Development Goal 3.8 explicitly calls for achieving UHC by 2030, including financial risk protection and access to quality essential health services (2).

However, global progress towards UHC has gone significantly off track. The 2023 Global Monitoring Report on Universal Health Coverage shows that although the global UHC service coverage index increased from 45 to 68 (out of 100) between 2000 and 2021, the pace of improvement has slowed markedly since 2015. In 108 countries, service coverage has not improved significantly since 2015, and some have even experienced regression. In 2021, 4.5 billion people globally (nearly half the world's population) still lacked access to essential health services (3). Faced with this serious situation, the WHO emphasises that the “fastest, most effective, and most equitable path” to UHC is through the PHC pathway, which can provide 90% of essential health service interventions (1).

### The quality dilemma of primary health care in low-resource settings

Despite broad consensus on the strategic importance of PHC, the quality of primary health care in low-resource regions globally, particularly in low- and middle-income countries and geographically dispersed rural and remote areas, remains deeply concerning. As synthesized from multiple reviews in Table 1, these quality challenges are multi-dimensional, spanning critical shortages in human resources and infrastructure, to severe deficiencies in service processes and governance coordination.

**Table 1:** Major challenges to primary health care service quality in low-resource settings globally: based on multiple review analyses.

Challenge dimension	Specific issues	Evidence sources
Human resources	Health worker shortages, high turnover; lack of professionals in rural areas; limited competency of primary care providers	(5, 8)
Service accessibility	Long travel distances (48% patients reported); long waiting times (74%); geographic isolation	(6, 7)
Medicines and supplies	Drug shortages (64%); supply chain disruptions; lack of basic equipment	(5, 6)
Infrastructure	Unreliable electricity; limited space; poor transport conditions	(5, 8)
Service quality	Poor staff attitudes (56%); low diagnostic accuracy; medication safety risks	(6, 8)
Financial affordability	Insufficient affordability (56%); high out-of-pocket expenditure	(3, 6)
Governance and coordination	Poor integration of PHC with multiple insurance schemes and vertical programmes; inadequate referral coordination	(8)
Health system support	Inadequate training; weak supervision and feedback; limited policy support	(8, 9)

Data in Table 1 are derived from a systematic review covering 52 LMIC health contexts 5 and empirical studies in multiple countries 5–9.

At the structural level (4), PHC in low-resource settings faces multiple shortages in human resources, infrastructure, medicines and supplies, and financial protection. A systematic review by Odhus CO and colleagues, synthesising 47 studies covering health contexts across 52 low- and middle-income countries, found that PHC quality is generally poor in low-resource settings, with limited resources often prioritised for tertiary care, even though PHC is the first and often only point of contact with the health system for nearly 80% of the population in LMICs (5). Key structural barriers identified in that review include inadequate health worker staffing and training, weak infrastructure (such as unreliable electricity and limited space), medicine shortages due to supply chain disruptions, and financial constraints (5). In rural and remote communities, structural problems are particularly acute. A mixed-methods study based on Tanzania's primary health system found that patients reported structural barriers including medicine shortages (64%), unaffordability (56%), and excessive travel distance (48%) (6). In rural and underserved communities, specialist shortages, limited diagnostic infrastructure, and geographic isolation persistently constitute core structural barriers to timely and accurate access to medical care (7). Health worker shortages and high turnover rates are also pervasive structural issues (8). Mwai D and colleagues further noted in their analysis of low-resource settings in Africa that even when professionals are posted to these areas, lack of supporting resources, low patient volumes, and cultural barriers often prevent them from delivering the high-standard services expected of their training (8). A scoping review systematically examining global PHC quality evidence corroborates these findings: human resources, legal and policy environment, infrastructure and facility conditions, and resource supply are the most common structural indicator categories in PHC service evaluation, with health worker shortages being a prominent challenge in both developed and developing countries (9). These structural deficits render primary care fundamentally compromised in two essential dimensions, safety and equity (10): shortages of personnel and equipment directly increase the risk of misdiagnosis and medication errors, while extreme resource imbalances between urban and rural areas mean that residents in remote areas are denied an equal starting point for quality care from the outset.

At the process level, multiple studies have identified problems including inadequate teamwork, lack of leadership, and weak supervision and feedback mechanisms (5–9). In the Tanzanian empirical study, 74% of patients reported excessive waiting times, and 56% reported poor staff attitudes (6). These problems reflect process deficiencies such as unreasonable PHC service workflow design and low-quality patient-provider interactions. At the level of specific service operations, core elements of the PHC process include health assessment and laboratory examination (diagnostic procedures) as well as health information provision, health education, and treatment (management procedures); however, these process elements are generally poorly implemented in low-resource settings, with medication safety hazards also being a prominent process-level problem (8). At the governance and coordination level, poor integration of PHC with multiple insurance schemes and vertical programmes, along with inadequate referral coordination, further undermines the continuity and coordination of service processes (8). In addition, inadequate training and the absence of supervision and feedback mechanisms are also systemic process-level issues in need of urgent resolution (7, 8). These process deficiencies place primary care under pressure across three dimensions: timeliness, patient-centredness, and efficiency. Long waiting times, indifferent staff attitudes, chaotic referral pathways, and duplicated efforts collectively constitute the most tangible quality dilemmas in patient experience (6–9).

At the outcome level, low diagnostic accuracy, poor chronic disease control, and low patient satisfaction are pervasive in PHC in low-resource settings (8). As the UHC monitoring data described above indicate (3), the global pace of service coverage improvement has slowed, and persistent coverage gaps remain. In cross-country comparisons on the outcome dimension, high-income countries demonstrate lower cause-specific mortality and hospitalisation rates, while developing countries face prominent challenges such as high tuberculosis mortality and geographic disparities in service quality (9). Low diagnostic accuracy and poor chronic disease control are direct consequences of insufficient effectiveness, low patient satisfaction reflects the absence of patient-centredness, and the vast quality disparities between regions are a full manifestation of the unfulfilled equity goal. The quality dilemma of PHC in low-resource settings is not a unidimensional deficiency, but a compound systemic problem in which structural resource scarcity and process-level operational deficiencies are intertwined and multi-layered (4–9).

### Quality challenges in primary care in China

China, as the world's largest developing country, also faces systemic challenges in primary care quality. Over the past decade, China has invested substantial resources in primary health care. However, evidence indicates that the quality of primary care remains far from ideal.

A 2024 study published in The Lancet Regional Health – Western Pacific used a standardised patient method to systematically assess service quality across five major types of primary care providers in China (village clinics, county hospitals, migrant worker clinics, urban community health centres, and online platforms). The study found that the overall quality of primary care in China is poor, with significant variation across provider types: community health centres performed relatively reliably in process quality, diagnostic accuracy, and correct prescribing, but village clinics, county hospitals, and migrant worker clinics lagged significantly behind. The study also found a positive correlation between physician qualifications and primary care quality, highlighting the urgency of deploying higher-quality professionals at the primary level (11).

At the human capital level, a large-sample 2023 survey covering 8,537 primary care physicians across eastern, central, and western China revealed that the competence rate for managing common and frequently occurring diseases was 82.1%, but competence rates for emergency critical care (50.6%), care coordination (56.7%), and traditional Chinese medicine services (52.7%) were relatively low. More noteworthy is the gap between the competence rate and the rate of meeting work needs: although competence rates among primary care physicians in central-western and rural areas were generally higher, the rate of meeting work needs was lower (12). This means that even when physicians possess a competency, they may still be unable to deliver expected service standards in practice due to resource scarcity, low patient volumes, or cultural barriers (8).

At the system level, primary care institutions in China face challenges including imbalanced service supply, substandard first-visit rates, and an irrational and ageing workforce structure. Only about 30% of China's health resources are distributed in townships and rural areas, with even more limited allocation in remote and rural regions (13). Uneven distribution of quality medical resources, insufficient professional competence of primary care physicians, outdated facilities, and inadequate application of digital technologies collectively constrain the service capacity of primary care institutions (14). In low-resource, geographically dispersed rural and border areas, these challenges are particularly acute. Vast territories, scattered populations, shortages of quality medical resources, coupled with heavy workloads and limited clinical experience of primary care workers, constitute the double pressure on primary care in these regions (12, 13). Although China is classified as an upper-middle-income country (15), these regions exhibit a triple predicament: extreme geographic dispersion (fewer than 1.5 health workers per 1,000 population), frequent seasonal population mobility, and weak digital infrastructure. These features resemble, in several structural respects, primary care conditions in many low- and middle-income countries (LMICs) and fragile states, although important differences in financing, governance, and institutional capacity should be acknowledged.

### AI-assisted diagnostic systems: the promise of empowering primary care

Against this backdrop, AI-assisted diagnostic systems are expected to empower primary care and bridge competency gaps (14, 16). AI has the potential to partially alleviate the most acute human resource shortages in low-resource settings. Sub-Saharan Africa has only 0.3 physicians per 1,000 population, less than one-tenth of the OECD average (8); in rural Bangladesh, the physician gap exceeds tenfold, with over 75% of the rural population turning to informal providers as their first point of contact (7). In such contexts, the value of AI-assisted diagnostic systems lies not in replacing physicians, but in serving as a clinical force multiplier (17), helping existing primary care workers make more accurate clinical judgments with limited training and support. AI's true contribution lies not in the novelty of the technology itself, but in whether it can increase the availability of safe, effective clinical support under resource constraints (16), a response to the structural human resource shortages in low-resource settings.

AI's empowering potential is more direct. As noted above, PHC in low-resource settings faces systemic process-level problems including low diagnostic accuracy, prominent medication safety hazards, and inconsistent guideline adherence (5–8). Primary care clinicians often face complex diagnostic and treatment decisions without timely supervision from senior physicians (8). AI-assisted diagnostic tools have demonstrated significant potential in improving diagnostic accuracy, rationalising prescribing behaviour, and enhancing chronic disease management efficiency among primary care workers (14, 16, 18). An umbrella review covering 26 studies found that AI-driven diagnostic tools were associated with improved diagnostic accuracy, reduced turnaround times, and enhanced service accessibility through mobile and telemedicine applications (7). A cluster-randomised trial from 16 primary care facilities in Kenya provides even more robust evidence: covering over 9,600 patients, the study showed that AI-assisted clinicians outperformed the control group in both diagnostic documentation and treatment plan quality (19). In a pilot study in Bangladesh, an AI system achieved an overall diagnostic accuracy of 94.7% across 287 clinical encounters (17). These findings suggest that AI can help primary care workers reduce diagnostic errors and non-standardised treatment by providing real-time diagnostic suggestions and treatment references, bringing service processes closer to evidence-based standards. At the same time, AI-assisted telemedicine and mobile health applications have the potential to shorten diagnostic waiting times and reduce unnecessary repeat visits (16), offering opportunities for improvement in the Timely and Efficient dimensions (10).

AI's ultimate value should be reflected in improved patient health outcomes. Although large-scale, prospective validation from low-resource settings remains limited (4), preliminary evidence suggests that AI-assisted decision support systems can reduce misdiagnosis and inappropriate treatment in primary care. A real-world study showed that AI assistance reduced diagnostic errors by 16% and treatment errors by 13% (16). Theoretically, improvements in diagnostic accuracy and treatment standards at the process level are expected to translate into better chronic disease control, reduced avoidable hospitalisations, and fewer patient safety incidents (8). On the Patient-centred dimension (10), AI tools, if developed through multi-stakeholder co-design, may also improve the quality of patient-provider communication and care coordination efficiency (5).

In LMICs, AI holds the promise of bringing step-change improvements to primary health care, but its impact will depend more on data infrastructure, governance systems, human resource capacity, and safety evaluation mechanisms than on the novelty of the models themselves (16).

In China, AI-assisted diagnostic systems have been progressively deployed in primary care facilities in some regions. The National Health Commission continues to promote the application of medical AI-assisted diagnostic technologies at the primary level, providing diagnostic decision support for common and frequently occurring diseases to primary care general practitioners and village doctors. China's primary care general practitioner intelligent decision support system can provide differential diagnosis recommendations and treatment references based on patient symptoms, signs, and medical history (20). At the policy level, the national target is to fully integrate AI-assisted diagnostic tools into primary care facilities by 2030 (18).

### From technical deployment to clinical adoption: global implementation evidence and the persistent gap

However, technical deployment does not automatically translate into improved clinical practice. Translating AI systems from technical deployment to real-world clinical practice improvement remains a formidable challenge. Global evidence indicates that the actual clinical adoption rate of AI-assisted decision support systems falls far short of expectations (20).

In terms of barriers, an umbrella review covering 26 studies systematically identified core barriers to implementing AI diagnostic tools in rural and low-resource settings: limited digital infrastructure, gaps in provider training, data privacy concerns, and regulatory uncertainty (7). Other studies have noted that interoperability limitations affect over half of AI implementation projects, and approximately 35% of projects face clinician distrust of unsupervised systems (20). At the primary care level, systematic reviews have revealed additional barriers including concerns about changes to the patient-provider relationship (loss of empathy, connection, and trust), concerns about data privacy and quality and algorithmic bias, and suspicion of increased workload; facilitators include the potential to improve workflow and decision support, enhance equity and accessibility, and stakeholder co-design (16).

In terms of facilitators, strong organisational culture support, stakeholder engagement, education and training, leadership support, and clear policy guidance have all been shown to significantly enhance AI system adoption and sustained use (20). In rural and underserved communities, community trust, integration with existing health systems, and a supportive policy environment are key enabling factors (7).

Notably, current research on AI decision support system implementation is predominantly concentrated in high-income countries or urban healthcare settings (20). AI healthcare applications in LMICs face additional barriers, including data privacy concerns, unresolved data ownership issues, and economic constraints (16). The global AI divide risks further exacerbating inequalities in health, education, and governance (16). Furthermore, scholars have observed that starting from technology without understanding actual system needs often leads to poor implementation outcomes (20). For low-resource settings, whether AI can become a catalyst for health equity in primary health care depends on whether digital infrastructure gaps, affordability, connectivity, and human resource training are addressed (16).

In the Chinese context, AI implementation in primary care faces distinct challenges. Studies have pointed out that China's primary care system faces persistent challenges including uneven resource distribution, general practitioner shortages, and increasing chronic disease burden (14); even where AI systems have been introduced, challenges remain including limited model explainability, potential erosion of humanistic care, accessibility inequalities, technical constraints, and data privacy concerns (18). Other studies have emphasised that AI applications still face multiple challenges including data quality, data security, and ethical norms (18, 20, 21). Regulatory approvals under controlled conditions typically confirm that AI systems meet baseline safety and accuracy standards, but once deployed in clinical settings, actual performance may vary considerably (20).

### The lack of multi-actor co-creation: a deeper root of the AI implementation gap

At its root, the AI implementation gap largely stems from a structural imbalance in the development ecosystem, a supply-driven model that dominates the entire process of AI system design, development, and deployment. Studies have noted that AI systems in healthcare often have high developmental precision yet fail to achieve meaningful clinical impact; the core of this last-mile problem lies in whether end-users recognize, accept, and act upon AI recommendations (22). Technology companies, research institutions, and policymakers constitute the core decision-making circle, while frontline clinicians, community health workers, patients, and the public (the end-users) have virtually no voice in the early stages of system development, including needs definition, functional design, and interface optimisation (23). Systematic reviews indicate that although participatory approaches are increasingly adopted in digital health innovation, they are primarily concentrated in the early design phase, with very limited participation in later implementation, audit, or governance stages (24). General practitioners have reported difficulties with workflow integration, concerns about loss of clinical autonomy, and lack of clarity on how AI systems fit into care pathways (23). User-centred design research demonstrates that understanding end-user approaches, intentions, and gaining critical insights is a key pathway to improving AI tool adoption (25).

Viewed through the Patient-centred dimension of the IOM model (10), this problem is particularly salient. Qualitative studies have found that patients and the public have important views on AI implementation and wish to participate in relevant discussions; patients believe AI tools should be developed with diverse patient input to ensure inclusivity and usability (22, 26). A Canadian national deliberative dialogue study also showed that participatory co-design and iterative implementation were most strongly advocated by multiple stakeholders as core strategies to overcome AI implementation barriers (27). The lack of multi-actor co-creation mechanisms, that is, the collaborative participation of technology suppliers, service providers, patients and the public, and policymakers throughout the AI system lifecycle, has been identified in multiple studies as a core barrier to AI implementation in primary care (22, 24, 27), undermining its relevance and acceptability in clinical practice.

### Application of implementation science theoretical frameworks in this study

Implementation science provides powerful theoretical tools for systematically identifying and explaining implementation gaps (28, 29). CFIR was first proposed by Damschroder and colleagues in 2009 (28) and later iteratively updated to CFIR 2.0 (29), and is currently one of the most widely applied frameworks in implementation science. CFIR organises implementation determinants into five core domains: Innovation, Outer Setting, Inner Setting, Individuals, and Implementation Process (28, 29). CFIR has been shown in multiple healthcare contexts to effectively guide qualitative data collection and analysis (30).

As CFIR, as a macro-level framework, cannot fully explain the complex mechanisms at the individual behaviour level (31), this study introduces the COM-B model and TDF for healthcare worker usage behaviour (30–32). The COM-B model explains the mechanisms of behaviour from three dimensions: Capability, Opportunity, and Motivation (31); TDF further disaggregates.

COM-B constructs into 14 theoretical domains (32). Previous studies have successfully integrated CFIR with COM-B&TDF to analyse cross-level influencing factors in implementation (33).

For the institutional and governance level, this study will examine how policy requirements, professional norms, legitimacy, and multi-actor collaboration shape implementation behaviour. Consistent with the study's overarching implementation-science framework, these institutional- and governance-level factors will be analysed within the outer-setting domain of the CFIR 2.0 framework rather than through a separate theoretical model.

### Research gaps and significance of this study

The following key research gaps currently exist: (1) Systematic mixed-methods studies on the implementation of AI-assisted diagnostic systems in primary care in low-resource countries, including African countries and even low-resource, geographically dispersed regions of China, remain scarce; (2) Implementation science theoretical frameworks have not been fully utilised to comprehensively identify and analyse influencing factors and their interaction mechanisms in AI implementation in primary care in low-resource settings (20); (3) Existing studies predominantly focus on quantitative surveys of technology acceptance, with insufficient illumination of the underlying institutional, organisational, and behavioural mechanisms (14, 18); (4) There is a lack of multi-actor co-creation research, particularly in China.

The significance of this study is threefold. First, implementation science significance: enriching the application of implementation science theoretical frameworks (particularly CFIR) in AI implementation research in low-resource, geographically dispersed settings. Second, public management significance: revealing the deep-seated technology-organisation-policy disconnection from institutional and governance perspectives (33, 34). Third, practice improvement significance: providing concrete, actionable strategy recommendations for optimising the implementation of AI-assisted diagnostic systems in primary care in low-resource settings (35). Fourth, governance model significance: from a multi-actor co-creation perspective, revealing the structural limitations of the supply-driven model in AI system development and implementation, and highlighting the value of collaborative participation of end-users (clinicians, community health workers, patients, and the public) throughout the system lifecycle (22–24, 27).

### Implications for AI governance in resource-constrained and geographically dispersed settings

Drawing on the implementation science and AI adoption literature reviewed above, we identified several candidate considerations that warrant empirical examination in this study, rather than being asserted as predetermined recommendations. These include: (1) whether infrastructure pragmatism (for example, the need for edge computing or offline functionality where connectivity and electricity are unreliable) influences uptake in pastoral and remote areas; (2) whether task shifting and incorporating AI operation into community health worker training reduces perceived workload barriers; (3) whether clear legal liability and accountability guidance affects clinicians’ willingness to act on AI outputs; and (4) whether participatory, human-centred design improves acceptance and use. These candidate considerations will be examined as research questions in Phases I and II and, where supported by the evidence, will be converted into formal strategies in Phase III through the Behaviour Change Wheel and Implementation Mapping.

## Research objectives

This study aims to systematically identify key influencing factors and their mechanisms in AI-assisted diagnostic system empowerment of primary care in low-resource settings. Specific objectives include:

1. To understand the perceptions, attitudes, and practical experiences of primary care clinicians, facility managers, and relevant stakeholders regarding the role of AI-assisted diagnostic systems in primary care;
2. To identify key factors influencing the application of AI-assisted diagnostic systems in primary care, covering institutional support, resource availability, organisational management, technical fit, collaborative relationships, and behavioural factors;
3. To survey primary care patients’ awareness, attitudes, trust, and acceptance of AI-assisted diagnosis, and to analyse the acceptability of AI-assisted diagnosis and its influencing factors from the patient perspective;
4. To develop a study-specific patient questionnaire informed by the patients’ perspectives identified in Phase I and by existing theory, and to identify and quantify patient-level acceptance of AI-assisted diagnosis and its influencing factors, including the mechanisms underlying acceptance;
5. To formulate candidate optimisation strategies and implementation pathway recommendations suitable for low-resource primary care contexts using behaviour change theory and implementation pathway design methods, and to develop an implementation framework whose transferability to similar resource-constrained settings will be explicitly assessed.

The target intervention defined in this study is the AI-assisted diagnostic system deployed or planned for deployment in primary care facilities in low-resource settings. The primary intended users of the systems under study are primary care clinicians and village doctors, who receive AI-generated diagnostic or clinical decision support at the point of care and retain the final clinical decision. The main clinical functions covered by this protocol include differential diagnosis suggestions, treatment references, risk alerts, and follow-up management prompts, implemented through systems such as clinical decision support systems, intelligent triage systems, medication support systems, and chronic disease follow-up management systems. Where participating facilities differ in their specific clinical functions, intended users, degree of autonomy, or level of human oversight, these differences will be documented and taken into account when interpreting barriers and facilitators and when assessing whether findings from different facilities concern comparable interventions. Throughout this protocol, we use the consistent term “AI-assisted diagnostic systems” to refer to the intervention under study.

### Stakeholder definition (7P principle)

This study adopts the 7P principle (36) to systematically define stakeholders:

Patients: Patients receiving services at primary care facilities and their family members. Within this category, we will prioritise the inclusion of older patients (aged 65 years and above), ethnic minority groups (particularly Mongolian-speaking pastoralists), and semi-nomadic populations, groups that are disproportionately affected by digital exclusion yet remain underrepresented in AI application research.

Providers: Frontline clinicians, including general practitioners, nurses, and public health physicians.

Purchasers: Organisations purchasing services on behalf of patients (e.g., health insurance agencies, medical consortium lead units).

Payers: Funding bodies paying for services (e.g., health insurance departments at various levels, financial departments).

Public: Community populations covered by primary care services.

Policymakers: Decision-makers at health administrative authorities at various levels.

Product suppliers: AI system vendors, technical support, and maintenance personnel.

Based on this definition, interview respondents in this study cover five core stakeholder categories: service providers (Category A), facility managers (Category B), external managers/policymakers (Category C), product suppliers (Category D), and patients (Category E). Patients (Category E) will participate both in qualitative interviews in Phase I (with numbers determined by saturation within the category) and in the patient survey in the quantitative phase.

## Methods and analysis

### Study design

This study adopts a sequential mixed-methods design (37), progressing through three phases, with findings from each phase informing the subsequent phase. This design maximises the complementary strengths of qualitative and quantitative research: the qualitative phase provides deep understanding and contextual sensitivity, the quantitative phase quantifies patient-level relationships and tests the pathways underlying patient acceptance of AI-assisted diagnosis, and the third phase facilitates translation from theory to practice. The overall study design framework is presented in Figure 1 and Table 2.

**Figure 1:**
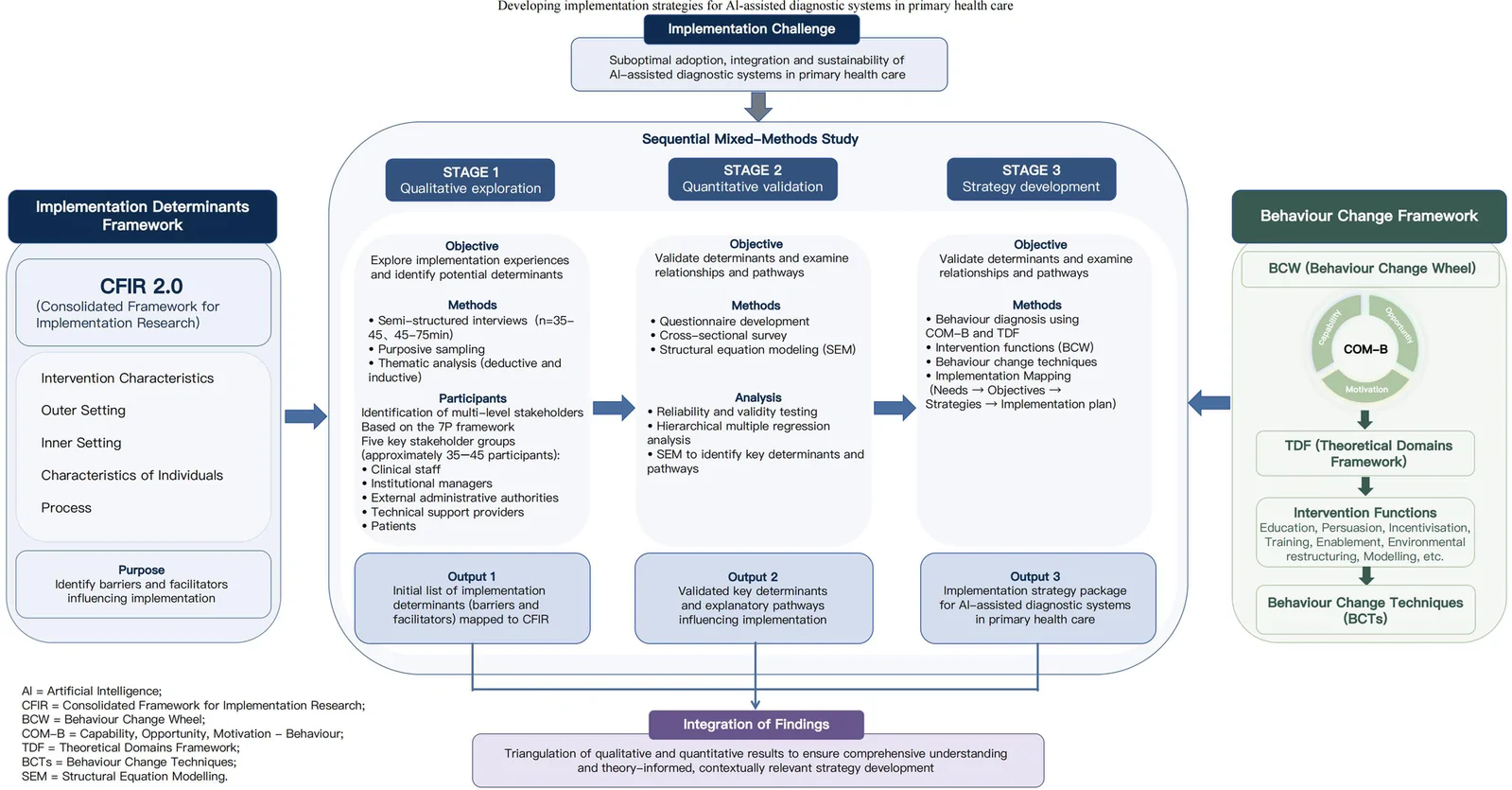
Conceptual framework of the sequential mixed-methods study.

**Table 2:** Overview of the three study phases.

Phase	Phase name	Main research method	Data sources	Expected outputs
1	Mechanism identification and contextual understanding	Semi-structured interviews, in-depth interviews, field observations	∼35-45 participants from 5 stakeholder categories	Key mechanism model, core variable set, interview guide
2	Quantification of key factors and path testing	Cross-sectional survey	∼500 patients	Quantitative path model, hypothesis testing results, questionnaire instrument
3	Optimisation pathway formulation	BCW + Implementation Mapping integration	Outputs from Phases 1 & 2 + theoretical integration	List of optimisation strategy recommendations, implementation pathway plan

### Theoretical framework

This study adopts a multi-level theoretical integration framework (Figure 1), specifically including:

Overarching implementation analysis framework: CFIR 2.0 (28, 29). CFIR 2.0's systematic nature enables coverage of a wide range of elements from technical characteristics to implementation environment. Under the CFIR framework, five core domains will be used for interview guide design, coding, and data interpretation: Innovation Characteristics (including source, evidence strength, relative advantage, adaptability, etc.,), Outer Setting (patient needs, external policies, resource environment, etc.,), Inner Setting (organisational culture, leadership engagement, available resources, training accessibility, etc.), Individual Characteristics (implementation-related knowledge and beliefs, self-efficacy, etc.), and Implementation Process (planning, engagement, execution, reflection and evaluation).

Behavioural explanation framework: COM-B + TDF (31, 32). For clinician usage behaviour, the COM-B model explains behavioural mechanisms from three dimensions: physiological/psychological Capability, physical/social Opportunity, and automatic/reflective Motivation (31). TDF further subdivides COM-B's six core constructs into 14 theoretical domains (e.g., knowledge, skills, beliefs, social influences, etc.), providing a more granular analytical tool for behavioural determinants (33).

Institutional and governance factors (including policy requirements, professional norms, legitimacy, and multi-actor collaboration (33, 34)) will be examined within the outer-setting domain of the CFIR 2.0 framework, which captures the external policy, regulatory, and relational context in which primary care facilities operate.

This study will not apply a closed theoretical mapping using selected constructs from the CFIR Individual Characteristics domain. Instead, the content of that domain will be referenced during interview guide design, and the analytical approach will follow an open coding to inductive thematic development to theoretical mapping pathway. Theoretical frameworks serve as tools for explaining and understanding research findings, rather than mechanical application to data.

To avoid theoretical overload, each framework has a clearly delineated role in this study: (1) CFIR 2.0 serves as the overarching implementation-analysis framework, guiding qualitative data collection (interview guide), coding, and interpretation of organisational and contextual determinants, including institutional- and governance-level factors within its outer-setting domain; (2) COM-B and TDF are used as behavioural-analysis frameworks, focused on the individual (clinician and patient) behavioural determinants identified within the CFIR structure; (3) the Technology Acceptance Model (TAM) is retained as a conceptual basis for patient acceptance: its core constructs (perceived usefulness and perceived ease of use) are operationalised as patient-level questionnaire measures and interpreted within the COM-B structure (perceived usefulness informing motivation and perceived ease of use informing capability), together with related acceptance constructs (e.g., trust, privacy concerns, and social influence) drawn from UTAUT and TPB; and (4) the BCW and Implementation Mapping are used exclusively in Phase III for strategy development. Table 3 summarises the correspondence between research objectives, stakeholder groups, data sources, constructs, analytical methods, and the role of each framework.

**Table 3:** Correspondence between research objectives, stakeholder groups, data sources, constructs, analytical methods, and theoretical frameworks.

Research objective	Stakeholder groups	Data sources	Core constructs	Analytical method	Framework role
Understand perceptions, attitudes, and experiences of AI-assisted diagnostic systems	Providers, facility managers, external managers/policymakers, product suppliers, patients	Phase I interviews, documents, field observations	CFIR 2.0 domains; COM-B dimensions; TDF domains	Thematic analysis with theoretical mapping	CFIR 2.0 (overarching); COM-B/TDF (behavioural)
Identify key factors influencing implementation (institutional, organisational, technical, behavioural)	Providers, facility managers, external managers/policymakers, product suppliers	Phase I interviews, documents, field observations	CFIR 2.0 constructs; COM-B/TDF domains	Inductive theme development → deductive theoretical mapping	CFIR 2.0; COM-B/TDF (institutional and governance factors examined within CFIR 2.0 outer setting)
Survey patients’ awareness, attitudes, trust, and acceptance of AI-assisted diagnosis	Patients (Phase II survey)	Phase II patient questionnaire	Awareness, trust, perceived usefulness, perceived ease of use, privacy concerns, perceived risk, social influence	Descriptive statistics; hierarchical regression	TAM/UTAUT/TPB (item sources); CFIR-derived constructs
Develop survey instrument and test hypothesised pathways	Patients (Phase II survey)	Phase II patient questionnaire	CFIR-derived patient-perceived contextual factors; COM-B mediators (capability, opportunity, motivation); acceptance	CFA; SEM; bootstrap mediation	COM-B (mediating mechanism); CFIR 2.0 (construct derivation)
Formulate optimisation strategies	All stakeholders; expert review panel; Community Advisory Board	Outputs of Phases I–II; expert/stakeholder review	Behavioural barriers mapped to COM-B; intervention functions; strategy criteria (feasibility, acceptability, impact, sustainability, equity)	BCW diagnosis; Implementation Mapping; modified Delphi consensus	BCW; Implementation Mapping (Phase III only)

### Study setting

This study is set in the Inner Mongolia region of China. Inner Mongolia Autonomous Region is located in northern China, covering approximately 1.18 million square kilometres with a sparse population averaging only about 20 people per square kilometre, and in the sparsest pastoral areas, fewer than 1 person per square kilometre. The region encompasses diverse ecological types, including agricultural, pastoral, and agro-pastoral areas, with pure pastoral banner counties and agro-pastoral banner counties accounting for a significant proportion, and pastoral residents exhibiting seasonal mobility patterns. This extreme geographic dispersion poses severe challenges to health service accessibility: the average service area per health facility is 275 square kilometres, 5.5 times the national average. Although the total number of health facilities in the region exceeds 26,000, health resources are extremely unevenly distributed, with primary care facilities accounting for only 32.4% of the region's health workforce while serving the vast majority of the region's land area. Thus, Inner Mongolia, with its pastoral and agro-pastoral areas exhibiting extreme geographic dispersion, low population density, sparse allocation of health workforce, and seasonal population mobility, presents structural characteristics that resemble, in several respects, primary care conditions in LMICs and other geographically marginalised settings, while acknowledging differences in financing and governance, making it an suitable natural laboratory for studying AI-assisted diagnostic system implementation mechanisms in resource-constrained, geographically dispersed settings. Objective indicators of resource limitation at the selected facilities (including health workforce density (the region has fewer than 1.5 health workers per 1,000 population), the average service area per health facility (275 km^2^, 5.5 times the national average), availability of diagnostic equipment, network and power connectivity, and distance to referral centres) will be documented systematically at site recruitment and used both to characterise the study setting and to support transferability assessments (38).

## Study phase I: qualitative data collection

### Study objective

The primary aim of this phase is to systematically explore the multi-level implementation experiences of AI-assisted diagnostic systems in primary healthcare settings within low-resource areas, and to comprehensively identify the potential determinants that influence system adoption and sustained use.

Specific objectives include: (1) to gain in-depth insights into the perceptions, attitudes, and practical experiences of key stakeholder groups (including primary care clinicians, facility managers, external administrators, patients, and technology suppliers) regarding the AI system, and to capture their usage behaviours and interaction patterns in real-world clinical contexts; (2) to systematically delineate the factors that facilitate or hinder implementation, drawing on the five core domains of the CFIR 2.0 with particular attention to the mechanisms operating at the levels of institutional support, organisational management, technological fit, resource availability, and cross-sectoral coordination; and (3) to construct a core variable framework for implementation success through triangulation of multiple data sources (in-depth interviews, document analysis, and field observations), thereby informing the design of the Phase II patient questionnaire and providing a multi-level understanding of implementation determinants.

### Study setting

The study setting covers 2 to 3 typical low-resource banner counties in Inner Mongolia Autonomous Region, selected to maximise regional diversity. Selection criteria include geographic type differences (agricultural, pastoral, agro-pastoral areas), duration of AI system deployment, level of usage activity, and regional representativeness. Specific sites include:

Institutional and policy level: Health commissions of each banner county;

Management and coordination level: Lead hospitals of each banner county’s medical consortium, regional heads of technology vendors;

Facility and implementation level: 2 to 3 township health centres/community health service centres and several village clinics per banner county.

### Study population and inclusion criteria

The study population includes five core stakeholder categories. Detailed inclusion criteria are presented in Table 4.

**Table 4:** Participant categories and inclusion criteria.

Code	Role category	Specific roles	Key information points	Sample size (guided by information saturation)	Inclusion criteria highlights
A	Frontline providers	General practitioners, clinical doctors, nurses, public health staff, IT officers	Usage experience, value perception, barriers, expectations	Determined by information saturation	Direct contact with AI system; cover high/medium/low frequency users; include those who are ambivalent or resistant
B	Facility managers	Directors/deputy directors of health centres/community centres, department heads	Workflow integration, training arrangements, organisational climate, assessment mechanisms	Determined by information saturation	Direct decision-making or implementation responsibility for AI introduction and daily management
C	External managers	Health Commission officials, medical alliance lead hospital managers	System design, policy direction, resource allocation, cross-organisational coordination	Determined by information saturation	Involved in AI project planning, budget allocation, or performance assessment formulation
D	Technical support	AI system vendors, operations and maintenance personnel, trainers	System deployment logic, technical support mechanisms, training implementation	Determined by information saturation	Direct experience with system deployment, maintenance, or training
E	Patients (qualitative phase)	Adult patients receiving services at primary facilities	Awareness, attitudes, trust, acceptance	Determined by information saturation	Age ≥18 years, able to complete questionnaire independently or with assistance,with consideration of gender, age and ethnic minority status.
	Patients (quantitative phase)			500 (survey)	Age ≥18 years, able to complete questionnaire independently or with assistance

### Sample size and sampling strategy

The qualitative phase will recruit participants across the five core stakeholder categories (service providers, facility managers, external managers/policymakers, product suppliers, and patients). Consistent with the information-saturation principle, the number of participants in each stakeholder category will not be fixed in advance; recruitment will continue within each category until thematic saturation is reached and no new meaningful themes emerge.

The sampling strategy primarily employs purposive sampling, supplemented by maximum variation sampling and snowball sampling. Maximum variation sampling ensures coverage of different geographic types, facility levels, roles, usage levels (representativeness of high-, medium-, and low-frequency users), and implementation stage differences; snowball sampling is used to reach key informants who are difficult to contact directly. Saturation will be assessed separately within each stakeholder category or analytic stratum rather than across the combined sample, because the stakeholder groups are heterogeneous and differ in their perspectives. If saturation is reached in one category while other categories remain unsaturated, recruitment will continue for the latter categories; conversely, additional participants may be sought in a category if preliminary analysis suggests that new themes are still emerging. Any decision to extend recruitment in a category will be based on team discussion and documented justification.

In addition, for the patient survey, we will employ proportionate stratified sampling to ensure adequate representation of older patients, Mongolian-speaking users, and families residing in remote pastoral areas. Field researchers will collaborate with village-level health workers to precisely identify and reach these underserved subgroups through home visits or community gathering points established during seasonal health promotion activities.

### Data collection methods

#### Semi-structured in-depth interviews

Semi-structured interviews will serve as the primary data source for the qualitative phase. The interview guide will be developed based on CFIR 2.0 and COM-B&TDF frameworks. To meet COREQ reporting requirements (39), the research team will document interviewer qualifications and the process of establishing rapport with respondents.

The mapping between core constructs in the interview guide and corresponding interview questions is illustrated by the example coding framework in Table 5.

**Table 5:** Illustrative example of a qualitative coding framework for thematic analysis (hypothetical).

Emergent theme	Theme definition	Mechanism explanation	Illustrative anchor (hypothetical, for demonstration only)	CFIR mapping	COM-B&TDF mapping
Perceived value discrepancy	Subjective assessment of whether AI system truly helps clinical work	Low perceived value → low use intention → system idleness	“This system gives suggestions similar to what I think, sometimes slower than my own judgment.”	Innovation Characteristics: Relative Advantage	Motivation: Reflective motivation
Time burden and workflow conflict	AI system adds extra steps, mismatched with existing workflow	Time-consuming → abandonment → perfunctory clicks	“Patients are already many; opening this system requires extra clicks, delays work.”	Inner Setting: Workflow; Innovation Characteristics: Complexity	Opportunity: Physical opportunity
…	…	…	…	…	…

This table presents a hypothetical coding framework for illustrative purposes only. The themes, definitions, and quotations shown are hypothetical examples intended to demonstrate how coding and theoretical mapping will be performed; they do not represent study findings or participant data.

Interviews are expected to last 45−75 min. Interviews will be conducted by research team members as one-on-one in-depth interviews in quiet, private settings that do not disrupt respondents’ normal work (e.g., meeting rooms, consultation rooms at facilities). For respondents in remote areas or vendor staff, interviews may be conducted via secure online conference platforms when necessary. Respondents will receive informed consent forms in advance; interviews will be audio-recorded in full with supplementary detailed interview notes. Interview memos will be written immediately after each interview, documenting on-site impressions, non-verbal information, and preliminary analytical thoughts.

#### Document analysis

The research team will systematically collect and review the following types of documents: project application materials/implementation plans (to understand the rationale, objectives, and planning of AI system introduction); training notices, training materials, sign-in records (to assess training coverage and quality); system operating instructions, job flowcharts, usage specifications (to understand system functionality and its fit with workflows); performance assessments, supervision records, meeting minutes (to analyse management commitment and assessment mechanisms); relevant policy documents, informatisation documents, resource guarantee documents (to assess the institutional environment and resource support). Document analysis will be recorded in thematic summary format, with key information extracted to supplement and validate interview findings.

#### Non-participant field observations

Where conditions permit, short-term non-participant observations will be conducted in outpatient services, chronic disease follow-up, and public health services at selected primary care facilities, focusing on: at what points the AI system is opened and closed, who operates it, whether it affects service rhythm, under what circumstances it is skipped or bypassed, and what specific obstacles are encountered. Observations will not involve participation in clinical care, recording of identifiable patient information, or disruption of work processes.

### Qualitative data quality control

Multi-source data triangulation will be employed, triangulating interview data (different roles), document analysis, and field observations to enhance the reliability of findings. Weekly coding discussions and theme calibration meetings will be held to ensure consistent understanding of coding rules and theme development. The research process will maintain a complete audit trail, including interview logs, detailed codebooks, memos, and theme version iteration records. Research team members will regularly document their pre-existing assumptions and personal reflections on AI, primary care, and implementation issues to mitigate the influence of researcher bias on data interpretation. The process will clearly document when thematic saturation is reached and which themes have the strongest evidence.

## Study phase II: patient quantitative data collection

### Study objective

This phase aims to identify and quantify, at the patient level, patients’ acceptance of AI-assisted diagnosis and its influencing factors, using a cross-sectional survey administered to a large sample of primary care patients. The patient questionnaire will be developed after Phase I, based on the factors that patients themselves perceive as relevant to acceptance (identified from the Phase I patient interviews) and on existing theory (including the Technology Acceptance Model). Phase II is not intended to verify the qualitative findings of Phase I, and the two phases are not treated as mutually validating data sources; rather, Phase II extends Phase I by quantifying, from the patient perspective, the patient-level component of the implementation problem. We note that Phase I identifies determinants at multiple levels (individual, organisational, and external); of these, only those that can be meaningfully operationalised as patient-level perceptions or experiences (e.g., awareness, trust in the primary healthcare system, perceived usefulness, perceived ease of use, privacy concerns, perceived risk, and social influence) will be measured in the patient survey. Organisational-level determinants (e.g., implementation climate, leadership engagement, workflow integration, incentives, and provider use behaviour) are not directly measurable through patient self-report and are therefore examined qualitatively in Phase I and incorporated into Phase III strategy development.

Specific objectives are:

1. to develop and validate a study-specific patient questionnaire after Phase I, informed by the patients’ perspectives identified in the Phase I interviews and by existing theory, measuring patients’ acceptance of AI-assisted diagnosis, and to identify and analyse the principal factors influencing patient-reported acceptance;
2. to test the direct and indirect pathways among the patient-level determinants (awareness, trust in the primary healthcare system, perceived usefulness, perceived ease of use, privacy concerns, perceived risk, and social influence) and patient acceptance of AI-assisted diagnosis via structural equation modelling, quantifying the direction and strength of effects, including the hypothesised mediating role of patient-level capability, opportunity, and motivation;

### Study design

This phase adopts a cross-sectional survey design to systematically collect quantitative data on primary care patients’ awareness, attitudes, trust, and acceptance of AI-assisted diagnosis. The reporting of this cross-sectional study will strictly adhere to the STROBE Statement for Cross-sectional Studies (Strengthening the Reporting of Observational Studies in Epidemiology: checklist for cross-sectional studies). The study protocol has been designed and will be reported in accordance with each checklist item, covering sampling methods, variable definitions, data sources, bias control, and statistical analytical strategies, to ensure transparency of the research process and reproducibility of the findings.

### Study population and inclusion criteria

Adult patients receiving services at primary care facilities. Specific inclusion criteria are presented in Table 4.

### Questionnaire development and design

Based on core factors identified in the qualitative phase, survey questionnaires will be developed:

Patient Questionnaire: Measures primary care patients’ awareness, attitudes, trust, and acceptance of AI-assisted diagnosis, and analyses the acceptability of AI-assisted diagnosis in primary care and its influencing factors. Patient questionnaires will be distributed on-site by researchers or with assistance from facility staff, and completed with researcher explanation to alleviate potential comprehension difficulties. Scale items will draw on validated instruments and be locally adapted based on key themes from the qualitative phase, using 5-point Likert scales. Table 6 summarises the core variables and their corresponding measurement approaches. The table also presents the theoretical grounding, number of items, scale type, and planned analytic role for each variable.

**Table 6:** Core variables, measurement instruments, and theoretical grounding for the patient survey.

Variable type	Variable name	Theoretical grounding	Operational definition	Instrument/source	No. of items	Scale type	Analytic use
Outcome	Patient acceptance of AI-assisted diagnosis	COM-B (Motivation, reflective)	Overall willingness to accept or use AI-assisted diagnostic suggestions during primary care consultations	Adapted from Davis (1989) TAM behavioural intention subscale; Venkatesh et al. (2003) UTAUT	4–6	5-point Likert (1 = strongly disagree to 5 = strongly agree)	Dependent variable in regression
Independent	Awareness of AI-assisted diagnosis	CFIR 2.0 (Knowledge & Beliefs)	Patient's level of understanding of AI applications in healthcare	Self-developed based on qualitative themes; informed by Bansal et al. (2024) digital health literacy measures	3–4	5-point Likert	Predictor in regression
	Perceived usefulness	TAM (PU)	Extent to which patient believes AI-assisted diagnosis improves accuracy, efficiency, or quality of care	Davis (1989) Perceived Usefulness scale (culturally adapted Chinese version)	4	5-point Likert	Predictor in regression
	Perceived ease of use	TAM (PEOU)	Patient's subjective perception of how easy it is to understand or use AI-generated diagnostic information	Davis (1989) Perceived Ease of Use scale (culturally adapted Chinese version)	4	5-point Likert	Predictor in regression
	Trust in the primary healthcare system	CFIR 2.0 (Inner Setting)	Overall confidence in the reliability of primary care providers and institutions	Adapted from Hall et al. (2001) Physician Trust Scale; Thompson et al. (2022) primary care trust measure	4–5	5-point Likert	Predictor in regression
	Privacy concerns	CFIR 2.0 (Patient Needs) / COM-B (Opportunity)	Worries about security and confidentiality of personal health data used by AI systems	Adapted from Bansal et al. (2010) Privacy Concerns Scale; Smith et al. (2011) Information Privacy Concerns	3–4	5-point Likert	Predictor in regression
	Perceived risk	COM-B (Motivation, automatic)	Perceived likelihood of negative outcomes (e.g., misdiagnosis, delayed treatment) from AI-assisted decisions	Adapted from Cunningham et al. (2024) AI health risk perception scale	3	5-point Likert	Predictor in regression
	Social influence	TPB / UTAUT (Social Influence)	Attitudes or expectations of family, friends, or community members regarding use of AI in the patient's care	Adapted from Venkatesh et al. (2003) UTAUT social influence subscale	3	5-point Likert	Predictor in regression
Covariates	Age	–	Patient's actual age in years	Single self-report item	1	Continuous; categorised as ≤44 / 45–64 / ≥ 65 years	Control variable; subgroup grouping
	Gender	–	Patient's biological sex	Single self-report item	1	Binary (male/female)	Control variable; subgroup grouping
	Ethnicity	–	Patient's ethnic group	Single self-report item	1	Categorical (Han/Mongolian/other)	Control variable; subgroup grouping
	Education level	–	Highest level of formal education completed	Single self-report item	1	Categorical (primary or below / junior high / senior high or technical secondary / college or above)	Control variable; subgroup grouping
	Geographic area type	–	Urban-rural functional type of the patient's county of residence	Based on sampling stratification	1	Categorical (agricultural / pastoral / agro-pastoral)	Control variable; subgroup grouping
	Prior digital technology experience	–	Smartphone use frequency and prior experience with online health platforms	Self-developed	2–3	Smartphone frequency: 4-point Likert; online health use: binary+frequency	Control variable; subgroup grouping
Mediator	Capability (patient-level)	COM-B	Patient's understanding of and ability to make sense of AI-assisted diagnosis information	Self-developed based on qualitative themes; informed by digital health literacy measures	3–4	5-point Likert	Mediator in SEM
	Opportunity (patient-level)	COM-B	Patient's access to digital devices, internet, and social support needed to engage with AI-assisted services	Self-developed based on qualitative themes	3–4	5-point Likert	Mediator in SEM
	Motivation (patient-level)	COM-B	Patient's perceived usefulness, trust, and positive attitudes toward AI-assisted diagnosis (reflective); perceived risk and privacy concerns (automatic)	Adapted scales as listed above; self-developed items	4–6	5-point Likert	Mediator in SEM

All instruments will be adapted or validated for the target population as described in the “Questionnaire development and validation” section, and a pilot test (*n* = 30–50) in an independent sample will be conducted prior to full administration to assess reliability and validity.

TAM, technology acceptance model; UTAUT, unified theory of acceptance and use of technology; TPB, theory of planned behavior; CFIR 2.0, Consolidated Framework for Implementation Research (version 2.0); COM-B, capability-opportunity-motivation-behaviour model.

Questionnaire development and validation. The patient questionnaire is a study-developed instrument that is distinct from the qualitative coding framework: Table 5 is an illustrative coding framework for the Phase I thematic analysis (showing how theoretical constructs relate to preliminary codes), not a questionnaire scale or a validated measurement instrument, and the constructs and example items shown there do not represent the final questionnaire content. The questionnaire will be designed after Phase I is completed, rather than assuming that all measurement instruments are already fixed or culturally adapted. Its items are informed by the factors that patients themselves perceive as relevant to acceptance (identified from the Phase I interviews, which involve multiple stakeholder groups with particular attention to the patient perspective on acceptance, cognition, and intention to use) and by existing theory, including the Technology Acceptance Model (TAM). Specifically: (1) instruments that already have validated Chinese versions (e.g., the perceived usefulness and perceived ease of use subscales of the Technology Acceptance Model) will be adopted and adapted, and will be validated in the target population in combination with the Phase I findings; (2) items addressing study-specific constructs (e.g., awareness of AI-assisted diagnosis) will be developed from the Phase I qualitative themes and, where applicable, existing scales; and (3) content validity will be assessed by a panel of experts using the content validity index (CVI), with items of poor clarity or relevance revised or removed, and the measurement properties of the resulting instrument will be evaluated through exploratory/confirmatory analysis and internal-consistency indices. A pilot test (*n* = 30–50) in an independent sample will be conducted prior to full administration to assess reliability (Cronbach's *α*), item performance, and comprehension.

### Sampling procedure

To ensure representativeness and adequate coverage of underserved subgroups, a multistage stratified sampling strategy will be employed for the patient survey in Phase II.

#### First stage (facility selection)

This study will be conducted in Hohhot and Ordos cities in the Inner Mongolia Autonomous Region. Hohhot, the regional capital, is a capital-type metropolitan city, whereas Ordos is a resource-based prefecture-level city. The two cities differ substantially in administrative functions, economic development levels, and the allocation of primary healthcare resources. Notably, despite Ordos's GDP reaching RMB 612.2 billion in 2025, ranking first in Inner Mongolia and approximately 1.4 times that of Hohhot (RMB 427.8 billion), the availability of high-quality primary health resources in Ordos does not match its economic standing. This disparity underscores the limitations of economic affluence alone in ensuring equitable primary care capacity. Moreover, they are the prefecture-level cities in Inner Mongolia that have explicitly planned to systematically deploy AI-assisted diagnostic systems at the primary care level across their entire jurisdictions, rendering them of significant comparative research value. In addition, the two cities span both urban and rural (county/banner, including township and village) primary care settings, so the patient sample will include rural and remote facilities as well as urban community health centres, allowing the study to address its research question across the full continuum of primary care delivery. Resource-limitation indicators (workforce density, diagnostic capacity, connectivity, equipment availability, and distance to referral centres) will be collected at all participating facilities to characterise the resource context of each site.

Within these two cities, a multistage stratified random sampling strategy will be employed to select county-level administrative units and primary healthcare facilities. At the county level, all 18 county-level administrative units within the two cities constitute the sampling frame (Hohhot: 4 districts, 4 counties, and 1 banner; Ordos: 2 districts and 7 banners). These units will be stratified by urban–rural function into two strata: urban districts (6 in total) and county-level administrative units (12 in total, including counties and banners). Within each stratum, simple random sampling will be applied: 1 urban district will be selected from the urban stratum, and 2 county-level administrative units will be selected from the county/banner stratum, resulting in a total of 1 district and 2 counties/banners. At the facility level, all primary healthcare facilities within the selected 1 district and 2 counties/banners will constitute the sampling frame. Stratified sampling by urban–rural attributes will be employed: within the urban district, all community health service centres will be included; within the county-level administrative units, all township health centres and community health service centres within each jurisdiction will be included. Sampling at the county level and below will strictly adhere to randomisation principles.

#### Second stage (patient recruitment)

Within each selected facility, eligible adult patients aged ≥18 years who present for primary care services during the survey period will be consecutively invited to participate. To avoid selection bias, recruitment will be conducted on randomly selected weekdays and will cover different clinic sessions (morning and afternoon). Field researchers will collaborate with village health workers to actively reach and include patients from remote pastoral settlements through community health promotion events, home visits, or designated collection points during seasonal activities, thereby reducing the risk of underrepresenting geographically isolated and mobile populations.

### Sample size calculation

The quantitative phase plans to include a minimum of 482 valid patient responses. The sample size was calculated for the primary objective of estimating the proportion of patients who accept AI-assisted diagnosis. Using the cross-sectional study sample-size formula (*N* = [*Z*^2^ × *P* × (1−*P*)]/*d*^2^) with conservative assumptions: a 95% confidence level (*Z* = 1.96), the maximum-variance assumption for an unknown proportion (*P* = 0.5), and a small allowable error (d), a minimum of 382 valid responses is required. Anticipating that approximately 20% of returned questionnaires may be invalid or incomplete (e.g., due to patterned responding or excessive missing items), the required sample size was inflated accordingly, and the final target is set at a minimum of 482 valid responses (with recruitment of approximately 600 patients to achieve this). In addition, an *a priori* Monte Carlo simulation-based power analysis will be conducted in Mplus using the final measurement model and expected effect sizes (standardised path coefficients of approximately 0.2) before data collection, to confirm that the planned sample provides adequate power (≥ 0.80) for the structural paths of primary interest; if the simulation indicates insufficient power for specific paths, those analyses will be treated as exploratory or the target sample size will be revised accordingly.

### Data collection and quality control

Questionnaires will be administered exclusively through offline modes, combining paper questionnaires and researcher-assisted (face-to-face) completion, in order to include patients with limited digital literacy, connectivity, vision, or language barriers. No online questionnaire will be used. Distribution will be conducted through primary care facility management channels to ensure coverage of different facility types and geographic areas within the study region; however, to minimise selection and social-desirability bias, trained field researchers, rather than the clinical staff providing care, will administer or assist with questionnaire completion, and facility staff will only introduce the survey. All field researchers will receive standardised training before the formal survey and will follow a standardised administration protocol. Questionnaire completion will take place in a relatively private setting, free from interference by facility staff, village doctors, or other unrelated persons, and participants will be clearly informed that their responses will not affect their future medical care, to further reduce social-desirability bias. For participants who require assistance, researchers will provide neutral assistance according to a standardised script, without leading explanations or answering on behalf of the participant. Privacy will be protected by emphasising anonymity and confidentiality. Non-response (refusals and incomplete questionnaires) and questionnaire completion status will be recorded systematically (number of refusals, reasons where provided, and mode of administration) to enhance the transparency of the data collection process, and respondent characteristics (e.g., age, gender, ethnicity, education, and mode of administration) will be compared across the two administration modes (paper and researcher-assisted) to examine potential mode-related differences. The survey period is expected to be 1−2 months.

## Phase 3: exploratory strategy development

### Study objective

The goal of this phase is to translate the empirical findings of the preceding phases into a set of evidence-informed candidate implementation strategies for AI systems in low-resource primary care settings, integrating behavioural change theory with implementation pathway design. Phase III is an exploratory strategy-development phase; its purpose is not to implement or evaluate an intervention, and the strategies produced will require subsequent feasibility and effectiveness testing. Specific objectives include:

1. to integrate the qualitative and quantitative findings, identifying convergences and divergences between the two data streams and comparing differences across groups and regions, so as to furnish population-level empirical evidence to inform strategy development in Phase 3;
2. to systematically diagnose the key behavioural barriers identified in Phase 1 using the Behaviour Change Wheel (BCW) framework, mapping these barriers onto the capability, opportunity, and motivation components of the COM-B model, and aligning them with appropriate intervention functions;
3. to apply the systematic Implementation Mapping process to translate the behavioural diagnosis into actionable implementation strategies, covering the full continuum from strategy conception and stakeholder engagement to the development of specific action plans;
4. to preliminarily evaluate the feasibility, acceptability, and potential impact of candidate strategies against the real-world resource and regulatory constraints of primary care in Inner Mongolia, ultimately producing a set of context-tailored strategy recommendations and implementation roadmaps that are grounded in local practice and whose transferability to other resource-scarce and geographically dispersed settings will be assessed explicitly.

### BCW and implementation mapping methods

Based on the findings from the first two phases, the Behaviour Change Wheel (BCW) (31) and Implementation Mapping methods (40) will be used for integrated analysis. The BCW comprises three core elements: the COM-B behaviour model, intervention functions, and policy categories. By mapping the barriers identified in the qualitative phase onto the BCW analytical framework, specific intervention functions can be identified to address different types of barriers. Implementation Mapping provides a systematic process from needs assessment to implementation strategy development (29).

### Optimisation strategy formulation

The identified key behavioural problems will be matched against the diagnostic outputs of the BCW framework to develop an initial draft of intervention strategies. Priorities for improvement will be determined based on stakeholders’ preferences regarding the areas most in need of change, gathered during interviews. Following strategy formulation, candidate strategies will be assessed for feasibility and acceptability via a structured expert review, constrained by the practical resource conditions and institutional limitations of primary healthcare services in Inner Mongolia. This process will yield a list of optimised strategy recommendations tailored to the local context.

Expert and stakeholder review. The expert review panel will comprise approximately 8–12 members selected purposively to include implementation scientists, primary care clinicians and facility managers from participating sites, health-policy officials, technology-vendor representatives, and patient representatives from the Community Advisory Board. Experts will be selected based on their expertise in primary care, implementation science, health policy, or AI in healthcare, and their familiarity with the study context. A structured consensus method (a two-round Delphi process) will be used, and the process will be reported in accordance with the guidance for Conducting and REporting DElphi Studies (CREDES). Experts will rate each candidate strategy on standardised criteria: feasibility, acceptability, potential impact, sustainability, and equity. For strategies on which consensus is not reached after two rounds, a comprehensive judgement will be based on the second-round ratings and expert comments, and necessary dissenting views will be retained and documented. Each final strategy will be linked transparently to the specific empirical finding(s) (qualitative theme and/or quantitative path) from which it was derived, and this linkage will be recorded in the strategy formulation matrix. Because the focus of this study is to identify implementation barriers and facilitators and to formulate candidate strategies, Phase III does not include the actual implementation of strategies or the evaluation of intervention effectiveness, which will be addressed in subsequent research.

## Data analysis

This study adopts a sequential mixed-methods design, with distinct yet interconnected analytical strategies across the three phases. Phase I employs qualitative thematic analysis supplemented by theoretical mapping. Phase II uses descriptive statistics, hierarchical multiple regression, and structural equation modelling on the patient sample to test patient-level relationships. Phase III applies BCW diagnosis and Implementation Mapping for integrated analysis. Each phase is detailed below.

### Qualitative data analysis

Qualitative data obtained from Phase I interviews, document analysis, and field observations will be analysed using a hybrid deductive-inductive thematic analysis approach. This method preserves openness to data while maintaining theoretical sensitivity, making it particularly suitable for exploratory implementation research. The analytical process follows the four steps outlined below.

#### Step 1: deductively driven initial coding framework construction

Prior to data collection, the research team will deductively develop an initial coding framework (codebook) based on the five core domains of CFIR 2.0 (Innovation Characteristics, Outer Setting, Inner Setting, Individuals, and Implementation Process) and the COM-B model (Capability-Opportunity-Motivation). The mapping of these CFIR 2.0 domains to sample interview questions is presented in Table 7. The framework will also reserve a category for “emergent themes” to ensure inductive space. This coding framework will be iteratively revised as data collection and analysis proceed.

**Table 7:** CFIR 2.0 core constructs and sample interview question mapping.

CFIR 2.0 domain	Core construct	Sample interview question (for providers/managers)
Innovation characteristics	Relative advantage	“Compared with your usual diagnostic approach, what aspects of this AI system are better or worse?”
	Adaptability	“Can this system be adapted to your department's needs? Have you encountered any mismatches?”
	Complexity	“How convenient is this system to operate? Which steps are most time-consuming?”
	Evidence strength and quality	“Are you aware of any successful cases or research evidence from home or abroad for this AI system?”
Outer setting	External policy and incentives	“Has your region or organisation issued policies encouraging or requiring AI use? Are there any incentives?”
	Patient needs and resources	“What do you think patients think of this AI system? Do their attitudes affect your use?”
	External collaborative networks	“Besides your own unit, do the Health Commission, medical alliance, or technology company provide ongoing support? Is communication smooth?”
Inner setting	Implementation climate (leadership engagement)	“What is your unit's leadership attitude toward AI use? Are there specific promotion measures?”
	Available resources (training/technical)	“Is the training you received adequate? How are technical issues usually resolved? Response speed?”
	Communication and work mechanisms	“Has AI use been integrated into your daily workflow, e.g., shift handover, case discussions?”
Individual characteristics	Knowledge and beliefs	“Do you trust AI diagnostic suggestions? Have you ever worried about liability issues?”
	Self-efficacy	“Do you feel proficient in using this system? In what situations do you feel challenged?”
Implementation process	Planning and execution	“At the time of introduction, was there a clear implementation plan? Has it been followed in practice?”
	Reflection and evaluation	“Has your unit regularly evaluated AI system effectiveness? Are usage data fed back to providers?”

CFIR 2.0 includes 5 domains and over 40 constructs; only the most relevant core constructs are listed above.

#### Step 2: independent coding and iterative comparison

Two researchers will independently code the verbatim transcripts, assigning each meaningful data segment to the corresponding CFIR 2.0 construct or COM-B dimension. Segments that cannot be directly mapped onto existing constructs will be flagged and inductively coded as “emergent themes.” The coding results will be systematically compared; disagreements will be resolved through discussion, with a third researcher arbitrating if consensus cannot be reached. Analytical memos will be maintained throughout.

#### Step 3: deductive re-mapping of inductive themes

Following the completion of all coding, inductively derived emergent themes will be subjected to deductive reverse mapping against the CFIR 2.0 and COM-B theoretical constructs; that is, examining whether emergent themes can be explained by existing theoretical constructs; if not, they will be reported as additional “emergent themes” to preserve data richness. This deductive step leverages the explanatory power of established theories to illuminate the underlying implementation mechanisms behind each theme. The inclusion of themes in the final thematic report will not be determined by a fixed frequency threshold (e.g., mention by at least three participants). Instead, the decision will be based on a comprehensive consideration of the theme's conceptual importance, its relevance to the research question, its explanatory power, the position of the stakeholders reporting it and their professional or decision-making authority, the degree of corroboration across different data sources (interviews, field observations, and documents), and consistency across interviews as well as negative (deviant) cases. Consequently, even a theme raised by only one participant may be retained in the final analysis when that participant holds a special or key stakeholder identity and the issue carries important theoretical or practical significance. Theme frequency will be reported as descriptive information only, and will not be the sole basis for including or excluding a theme.

#### Step 4: coding consistency and quality control

The codebook will be continuously updated and refined, with a complete audit trail preserved. The criterion for thematic saturation will be the absence of new meaningful codes in three consecutive interviews. All qualitative data management will be conducted using NVivo 14 software.

To address the limited explanatory power of the CFIR framework at the individual behavioural level, this study simultaneously introduces the COM-B model (31) and the Theoretical Domains Framework (TDF) (32) in both interview guide design and subsequent analysis. Drawing on previous systematic reviews of AI/digital health technology adoption behaviours (16, 20, 22, 24) and the theoretical framework of this study, we hypothesise potential barriers and facilitators associated with primary care clinicians’ AI system use behaviour, preliminarily mapped onto the three COM-B dimensions and corresponding TDF domains. Table 8 presents this hypothesised mapping matrix, which will be empirically validated, revised, or expanded through inductive coding of actual interview data during analysis.

**Table 8:** Preserved mapping of potential barriers and facilitators to AI system use behaviour across COM-B components and TDF domains.

COM-B component	TDF domain	Potential barriers	Potential facilitators
Capability (Psychological)	Knowledge	Limited understanding of AI system functions and clinical value; unawareness of evidence base	Training programmes on AI clinical applications; clear communication of system capabilities and limitations
	Skills	Lack of operational proficiency; difficulty navigating interface; slow input speed	Hands-on training sessions; simplified user interface; on-site technical support
	Memory, Attention & Decision Processes	Complex workflows increase cognitive load; difficulty remembering system features under time pressure	Built-in clinical pathway integration; quick-reference guides; operational shortcuts
Opportunity (Physical)	Environmental Context & Resources	Unstable internet/power supply (especially in pastoral areas); insufficient hardware (computers/tablets); lack of dedicated consultation time for AI use	Edge computing/offline functionality; device provisioning by health authorities; protected time for AI use within consultation
Opportunity (Social)	Social/Professional Role & Identity	Fear of peer judgement or stigma (“AI users are lazy/incompetent”); AI perceived as incompatible with professional identity	Professional association endorsement; peer success stories; recognition as “innovation pioneer”
	Social Influences	Colleagues not using the system (normative pressure); supervisors indifferent to AI adoption	Visible leadership endorsement; team-based usage goals; peer learning communities
Motivation (Reflective)	Beliefs about Capabilities	Low self-efficacy in using AI; previous failed experience with digital tools; fear of making errors with AI assistance	Confidence-building through simulation training; gradual exposure (simple to complex tasks)
	Beliefs about Consequences	Fear of legal liability if following AI advice leads to error; concern that AI reduces diagnostic accuracy; worry that AI undermines clinical autonomy	Clear liability protection policies; AI positioned as “reference only”; evidence of error reduction from published studies
	Intentions	No intention to use AI unless mandated; perceived “not my decision”	Clear organisational expectations; integration of AI use into routine workflow as standard practice
	Optimism	Low perceived relative advantage compared to current practice; belief that “AI cannot replace clinical experience”	Demonstration of tangible benefits (time saved, improved documentation); real-world success stories from similar settings
Motivation (Automatic)	Reinforcement	No positive feedback or recognition for using AI; no consequences for non-use	Performance dashboards with usage metrics; “AI Power User” awards; integration into performance evaluation
	Emotion	Anxiety about technology; fear of being “replaced” by AI; frustration with system malfunctions	Reassurance of human oversight; emotionally supportive training environment; responsive technical support to reduce frustration

All hypothesised mappings will be empirically tested, revised, or expanded through inductive coding of Phase I interview data. TDF, Theoretical Domains Framework.

### Quantitative data analysis

Data obtained from the Phase II patient survey will be analysed using SPSS 26.0 (IBM Corp, Armonk, NY, USA) and AMOS 24.0 (or Mplus 8.0, depending on model complexity). The analytical strategy proceeds in three complementary stages: descriptive and difference testing, hierarchical regression, and structural equation modelling.

#### Descriptive statistics and difference analysis

Descriptive statistical analysis will be performed for all variables, calculating means, standard deviations, frequencies, and percentages, and examining the normality of data distribution. Independent samples t-tests (for two-group comparisons) or one-way analysis of variance (ANOVA; for three or more groups) will be used to test mean differences in core variables (AI awareness, attitudes, trust, privacy concerns, acceptance, etc.) across different demographic characteristics (e.g., gender, ethnicity, education level, geographic area type) and digital technology experience groups. Between-group comparisons for categorical variables will be conducted using *χ*^2^ tests.

#### Reliability and validity testing

Cronbach's *α* coefficient will be used to assess the internal consistency reliability of each scale, with *α* ≥ 0.70 considered acceptable. Confirmatory factor analysis (CFA) will be employed to evaluate the structural validity of the scales, examining factor loadings (standardised loadings ≥ 0.50 considered acceptable), composite reliability (CR ≥ 0.70), and average variance extracted (AVE ≥ 0.50). Model fit will be evaluated using the following indices: *χ*^2^/df < 3.00, CFI ≥ 0.90, TLI ≥ 0.90, RMSEA ≤ 0.08, and SRMR ≤ 0.08.

#### Hierarchical multiple regression analysis

Hierarchical multiple regression analysis will be used to identify factors influencing patient acceptance of AI-assisted diagnosis as the dependent variable. This method offers the advantage of assessing the incremental explanatory power of different blocks of variables on the dependent variable, specifically, whether core attitudinal and cognitive variables retain independent predictive effects on patient acceptance after controlling for basic demographic characteristics.

The regression analysis proceeds in two hierarchical steps. Step 1 enters demographic control variables, including age, gender, ethnicity, education level, geographic area type (agricultural/pastoral/agro-pastoral areas), and prior digital technology experience (e.g., smartphone use frequency, prior experience with online medical platforms), to eliminate confounding effects. Step 2 enters patient-level key factors inductively derived from Phase I qualitative interviews, including awareness of AI-assisted diagnosis, trust in the primary healthcare system, perceived usefulness, perceived ease of use, privacy concerns, perceived risk, and social influence (attitudes of family or friends) as core independent variables. All variables will be measured using Likert scales, with items based on themes identified in Phase I.

Model evaluation will examine multiple indicators. *R*^2^ change (Δ*R*^2^) will assess the incremental explanatory power of the Step 2 core independent variables on the variance of the dependent variable after controlling for demographic variables; a significant Δ*R*^2^ would indicate that patients’ subjective cognitive and attitudinal factors have an independent effect on AI acceptance beyond demographic background. Standardised regression coefficients (*β*) will be used to compare the relative strength of each predictor variable, with larger absolute *β* values indicating stronger effects on patient AI acceptance, and the sign indicating direction of effect. Adjusted *R*^2^ reflects the overall explanatory power of the model, and the overall model *F*-test determines whether the regression model is statistically significant.

Prior to entry into the regression model, variance inflation factor (VIF) will be used to test for multicollinearity among predictor variables, with VIF < 10 considered acceptable and VIF > 5 warranting attention. If severe multicollinearity exists (VIF > 10), consideration will be given to combining related variables or eliminating redundant variables.

#### Structural equation modelling

To test the hypothesised theoretical pathways, specifically how patient-perceived contextual factors (awareness, trust in the primary healthcare system, perceived usefulness, perceived ease of use, privacy concerns, perceived risk, and social influence) influence patient acceptance of AI-assisted diagnosis, both directly and through the patient-level mediating mechanisms of capability, opportunity, and motivation, covariance-based structural equation modelling (CB-SEM) will be employed using AMOS 24.0 or Mplus 8.0. CB-SEM was selected over partial least squares SEM because our objectives include confirmatory testing of hypothesised paths and formal mediation analysis, which require a full-information covariance-based approach and its associated global fit indices. SEM is particularly suited to this study for three reasons: (a) it enables simultaneous estimation of multiple direct and indirect pathways; (b) it accounts for measurement error in latent constructs, yielding less biased parameter estimates than regression-based path analysis; and (c) it allows formal testing of mediation hypotheses through bootstrap estimation of indirect effects. The full structural equation modelling analysis plan, prepared in accordance with the Journal Article Reporting Standards for structural equation modelling (JARS-SEM), is provided in the Appendix at the end of this protocol.

##### Model specification

The SEM will be constructed as a covariance-based structural model comprising three components:

Measurement model: Each latent variable (awareness, trust in the primary healthcare system, perceived usefulness, perceived ease of use, privacy concerns, perceived risk, social influence, and the three COM-B mediator constructs) will be indicated by its corresponding multi-item scale. The three COM-B constructs are operationalised at the patient level as follows: capability refers to the patient's understanding of and ability to make sense of AI-assisted diagnosis information (measured by items on comprehension and digital/health literacy related to AI); opportunity refers to the patient's access to the digital tools, internet, and social support needed to engage with AI-assisted services; and motivation refers to the patient's perceived usefulness, trust, and positive attitudes toward AI-assisted diagnosis (reflective motivation) as well as perceived risk and privacy concerns (automatic motivation). CFA will be conducted to confirm that the measurement model achieves acceptable fit prior to structural path estimation.

Structural model (direct effects): The model will estimate direct paths from the patient-level contextual variables (awareness, trust in the primary healthcare system, perceived usefulness, perceived ease of use, privacy concerns, perceived risk, and social influence) to the outcome variable (patient acceptance of AI-assisted diagnosis). These direct effects quantify the extent to which each predictor independently influences acceptance, controlling for all other variables in the model. The primary hypothesis (H1) is that these contextual variables jointly predict patient acceptance; secondary hypotheses specify the direction of each path (e.g., H1a: perceived usefulness positively predicts acceptance; H1b: privacy concerns negatively predict acceptance).

Structural model (indirect/mediation effects): Aligned with the COM-B framework as the hypothesised mediating mechanism, the SEM will test whether the relationships between the contextual variables and patient acceptance are mediated by the three patient-level COM-B dimensions (Capability, Opportunity, and Motivation); for instance, whether the effect of privacy concerns on acceptance operates through reduced motivation, or whether the effect of perceived usefulness operates through enhanced capability. The mediating hypotheses are specified *a priori* (e.g., H2: capability mediates the relationship between awareness/perceived ease of use and acceptance; H3: motivation mediates the relationships between perceived usefulness, trust, and acceptance; H4: opportunity mediates the relationship between digital access and acceptance). The significance of indirect effects will be tested using bootstrap resampling (5,000 resamples) with bias-corrected 95% confidence intervals; an indirect effect is considered statistically significant if the confidence interval does not include zero.

##### Model fit evaluation

Overall model fit will be assessed using a combination of absolute and incremental fit indices: *χ*^2^/df < 3.00, CFI ≥ 0.90, TLI ≥ 0.90, RMSEA ≤ 0.08, and SRMR ≤ 0.08. Model comparison will be conducted where theoretically justified (e.g., comparing a fully mediated model against a partially mediated model) using *χ*^2^ difference tests and information criteria (AIC, BIC). *Post-hoc* model modification will be applied only where theoretically plausible and supported by modification indices; any respecification will be documented, and final models will be cross-validated (e.g., via random split-half or a hold-out sample) where feasible.

##### Subgroup SEM analysis

To capture heterogeneity across different patient subgroups (as conceptualised in the theoretical framework), multigroup SEM will be conducted for older patients (aged ≥ 65 years), ethnic minority patients (Mongolian-speaking), patients with low education levels, and patients in remote pastoral areas. Prior to subgroup comparisons, measurement invariance of the latent constructs will be tested across relevant groups (configural, metric, and scalar invariance); meaningful group comparisons of path coefficients will be undertaken only where scalar (or at least partial metric) invariance is supported. Multigroup invariance testing will then examine whether path coefficients differ significantly across groups. This approach moves beyond the subgroup regression approach by simultaneously testing whether entire pathway structures, rather than individual coefficients, vary across subgroups. Results will inform the development of targeted, differentiated implementation strategies in Phase III.

##### Handling of missing data and non-normality

Questionnaires with more than 20% missing item responses on core scales, or with obvious patterned responding, will be excluded. For remaining item-level missing data, full-information maximum likelihood (FIML) estimation will be used within the SEM framework (Mplus), and multiple imputation will be applied for the regression analyses if the proportion of missingness exceeds 5%; the number of imputations will be based on the proportion of missing data. Variables with marked non-normality will be examined, and the Satorra–Bentler (MLR) robust estimator or appropriate transformations will be applied as needed.

##### Accounting for facility clustering

Because patients are recruited from multiple healthcare facilities and observations within a facility may be correlated, facility-level clustering will be accounted for. The Type = COMPLEX option in Mplus (or cluster-robust standard errors) will be used to obtain robust standard errors, and a multilevel sensitivity analysis will be conducted to examine whether the substantive conclusions change when facility-level random effects are modelled.

##### Primary and secondary hypotheses

The primary hypothesis is that the patient-level contextual variables predict patient acceptance of AI-assisted diagnosis (tested via hierarchical regression and the SEM direct effects). Secondary hypotheses concern the mediating roles of capability, opportunity, and motivation, subgroup differences in path coefficients, and the associations of demographic and digital-experience covariates with acceptance.

##### Integration with phase I qualitative findings

The Phase II results will be interpreted in conjunction with the qualitative themes from Phase I. The direction, magnitude, and significance of the patient-level relationships will be examined against the thematic framework following the convergent/complementary/divergent classification described in the mixed-methods integration section (Section III), and differences across groups and regions will be compared. Phase II is a deepening of Phase I at the patient level rather than a validation of the qualitative findings; this cross-phase interpretation ensures that the quantitative findings are contextualised within the perspectives and experiences of the study stakeholders and provides an empirical basis for Phase III strategy development.

### Mixed-methods integration analysis

Integration of qualitative and quantitative data will occur at the interpretive level, using side-by-side comparison and joint display methods (37).

#### Correspondence with the conceptual framework

The integration logic of this study is aligned with Figure 1 (Multi-level Theoretical Integration Analytical Framework). As illustrated in Figure 1, the five core domains of CFIR 2.0 (Innovation Characteristics, Outer Setting, Inner Setting, Individuals, and Implementation Process) constitute the macro-to-meso implementation context analytical dimensions, which are identified and explained through qualitative inductive development and theoretical mapping. The COM-B model (Capability-Opportunity-Motivation) serves as the mediating mechanism layer connecting contextual factors to individual behaviour, explaining why specific contextual factors facilitate or hinder use behaviour. The core task of mixed-methods integration is to examine how the qualitative findings (which span multiple stakeholder levels and identify organisational, contextual, and behavioural determinants) relate to the quantitative findings (which identify and quantify patient-level acceptance and its determinants). Phase II is not intended to verify the qualitative findings of Phase I; rather, it constitutes an in-depth extension of Phase I at the patient level, and the two phases together provide complementary evidence on the implementation problem. Where the two data streams diverge, the divergence will not be treated as indicating that one source is more valid; instead, possible sources of divergence (including differences in the populations and levels addressed by each phase and genuine differences in perspective across stakeholder groups and geographic areas) will be systematically examined, and differences across groups (e.g., by facility type, geographic area, or stakeholder category) will be compared. Integration therefore focuses on how the two sources of evidence jointly inform Phase III strategy development and provide an empirical basis for intervention research.

#### Construction of the concordance matrix

The research team will construct a joint display table, presenting in parallel the qualitative themes from Phase I (categorised by CFIR domains and COM-B dimensions) and the quantitative regression results from Phase II (significant/non-significant predictor variables and their standardised coefficients). These will be annotated according to three categories: (1) “Convergent”: quantitative results are consistent with qualitative themes in direction; (2) “Complementary”: quantitative results provide additional information not covered by qualitative themes, suggesting variables not captured by interviews; and (3) “Divergent”: quantitative results diverge from qualitative findings, prompting further examination of the reasons, including differences in the populations and levels addressed by each phase and genuine differences in perspective across groups or regions. Divergences will be interpreted as informative findings in their own right rather than resolved by privileging one source.

#### Divergence handling strategy

For areas of divergence, the research team will adopt a structured approach rather than assuming that either method is inherently more valid. Possible sources of divergence (including differences in the populations and levels addressed by each phase, measurement artefacts (e.g., item wording, social desirability, or recall bias), and genuine differences in perspective between stakeholders and patients) will be systematically examined by reviewing original interview materials, checking the accuracy of questionnaire item wording, and discussing measurement biases. Where a divergence reflects a genuine difference in perspective across levels (e.g., providers’ and patients’ differing views), it will be interpreted as an informative finding in its own right rather than resolved by privileging one source. Discussion of divergences will be reported as an important component of “methodological reflexivity” within the study findings.

#### Meta-inferences

Building upon the above integrative analysis, researchers will generate cross-phase meta-inferences that provide holistic answers to the study's core research questions and establish a complete evidence base for Phase III strategy development.

### Theory-to-practice translational analysis

Building on the integrated evidence from the preceding two phases, Phase III employs the Behaviour Change Wheel (BCW) and Implementation Mapping for systematic translational analysis. Phases I and II have already identified how CFIR contextual factors influence behaviour through COM-B mechanisms; Phase III builds on this foundation by selecting corresponding BCW intervention functions for the capability, opportunity, and motivation barriers that have been empirically identified, and developing candidate strategy packages through Implementation Mapping for subsequent research use.

#### BCW diagnosis

Key behavioural barriers jointly confirmed in Phases I and II (e.g., “perceived time burden”, “unclear accountability”, “inadequate training”) will first be diagnostically classified according to the three COM-B model dimensions: barriers attributable to Capability deficits (e.g., skill deficiencies, knowledge gaps) will be matched with “Training” and “Education” intervention functions; barriers attributable to Opportunity deficits (physical opportunity (poor hardware conditions); social opportunity (lack of institutional incentives)) will be matched with “Environmental Restructuring” and “Incentivisation” intervention functions; barriers attributable to Motivationdeficits (e.g., risk aversion, low perceived value) will be matched with “Persuasion” and “Modelling” intervention functions. This diagnostic process will produce a BCW diagnostic matrix.

#### Implementation mapping

The five-step Implementation Mapping process will then translate BCW diagnostic results into actionable strategy packages: (1) define implementation goals and expected outcomes (e.g., “increase daily AI system usage rate in primary care facilities to over 70%”); (2) identify performance objectives within the implementation process (e.g., “clinicians can independently operate the AI system”, “managers conduct regular data feedback”); (3) select theory-based behaviour change methods (e.g., “capacity-building training”, “peer modelling”, “performance feedback”); (4) translate methods into concrete implementation strategies (e.g., “monthly on-site operational drills”, “quarterly selection of AI usage champions”); and (5) assess strategy feasibility and acceptability, finalising the strategy list.

#### Feasibility assessment

Following strategy formulation, candidate strategies will be evaluated against the resource constraints and institutional boundaries of Inner Mongolia's primary healthcare services (including budget, available human resources, technical infrastructure level, and policy space) along multiple dimensions: intervention intensity, resource requirements, sustainability, political feasibility, and stakeholder acceptability. This will ultimately yield a priority-ranked list of context-sensitive strategy recommendations and an implementation roadmap.

### Software and reporting standards

All quantitative analyses will be conducted using SPSS 26.0 (IBM Corp, Armonk, NY, USA). Qualitative analyses will be managed using NVivo 14 (QSR International, Melbourne, Australia) for coding management. Reporting of the mixed-methods integration will follow the MMARS (Mixed Methods Article Reporting Standards) guidelines. The qualitative study report will adhere to the COREQ reporting standards (39) and the SRQR reporting standards (41).

## Study timeline

This study will run from April 2026 to April 2028, with an estimated duration of 2 years. The detailed schedule is shown in Figure 2.

**Figure 2:**
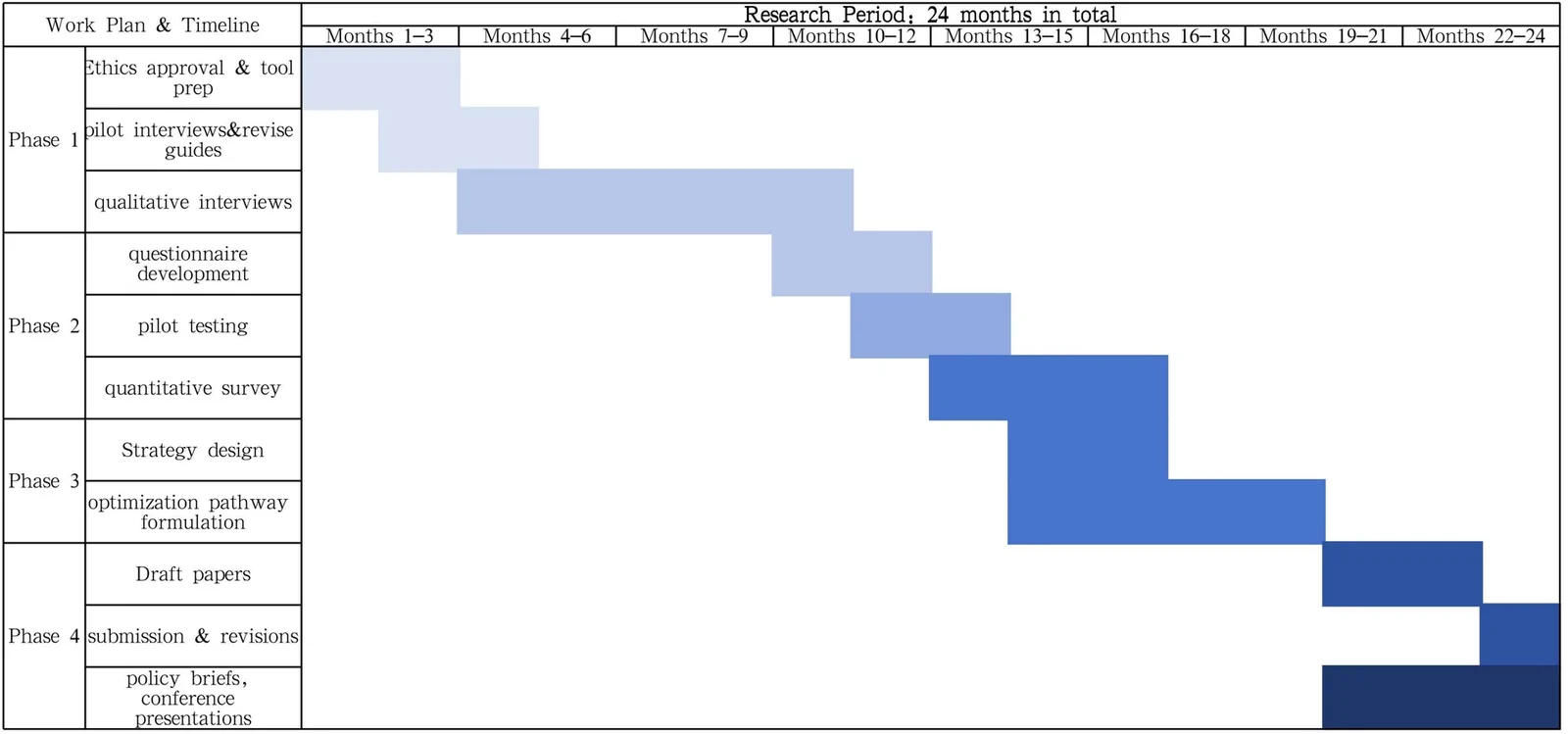
Research Gantt chart.

## Patient and public involvement (PPI) plan

Although patients and the public were not involved in the initial design of this protocol, we have developed a structured PPI plan to ensure meaningful engagement throughout the subsequent research phases. Prior to the quantitative survey (Phase 2), we will establish a Community Advisory Board (CAB) comprising 5–7 members, including: (a) 3–4 adult patients with recent experience of primary care services in the study banners (purposely including at least one elderly patient, one Mongolian-speaking participant, and one herder); (b) 1–2 family caregivers; and (c) 1 community health leader from a participating township health centre. The CAB will be convened via two in-person meetings (facilitated by a bilingual researcher with interpretation support) and, if needed, follow-up telephone consultations. The CAB's roles will be threefold: first, to review and culturally adapt the patient questionnaire, ensuring that language, concepts, and response options are comprehensible and locally appropriate; second, to provide contextual interpretation of preliminary qualitative findings from Phase 1, helping the research team to validate emergent themes and avoid misinterpretation of local practices; and third, to co-develop plain-language summaries of the study results for dissemination back to participating communities. CAB members will receive honoraria (approximately 300 RMB per meeting) and reimbursement for travel expenses as compensation for their time and contributions. All PPI activities will be documented in a dedicated PPI log, and reporting will follow the GRIPP2 short-form guidelines to ensure transparency.

## Ethics and dissemination

### Ethics approval

This study involves human participants (semi-structured interviews, patient questionnaires, and field observations) and has received formal ethical approval from the Medical Ethics Committee of Inner Mongolia Medical University (approval number: YKD202602099). All participants will provide written informed consent prior to any data collection. Given the relatively limited implementation science and AI research expertise in the Inner Mongolia region, protecting participant anonymity and privacy is a key focus of this study. The risk control measures adopted by the research team are summarised in Table 9.

**Table 9:** Summary of ethical and data protection measures.

Risk category	Specific risk	Protective measures	Reporting/handling mechanism
Identification risk	Small sample size, direct quotes may reveal identity	① Code instead of names; ② Remove institution and specific event details; ③ Quotes labelled only by category code	Anonymisation review during transcription
Psychological discomfort	Recalling negative experiences or exposing work problems during interviews	① Clearly state right to withdraw; ② Do not probe sensitive details; ③ Maintain neutral stance	If discomfort occurs, stop interview and provide mental health resources
Data breach	Unauthorised access or loss of electronic data	① Encrypted storage + dual backup; ② Paper materials locked; ③ Access permission Hierarchy	Report to PI within 24 h and notify ethics committee
Information misuse	Research results taken out of context for accountability	① Results reported in aggregate; ② Policy reports only offer system-level recommendations	Internal review before submission
Involuntary participation	Employees feel obliged to participate	① Informed consent emphasises voluntariness; ② Invitation through third party, not direct supervisor; ③ Can withdraw anytime	Withdrawal does not affect job evaluation

#### Protocol registration

Because PROSPERO does not accommodate primary mixed-methods studies of this kind, this protocol will be prospectively registered on the Open Science Framework (OSF) prior to the start of participant recruitment; the registration URL and date will be provided in the final study report and in the published protocol.

## Specific measures include

First, all interview records, transcripts, and coding files will use numbers instead of names and specific institutional names. Electronic data will be stored on encrypted devices and in restricted-access storage spaces; paper materials will be kept in locked cabinets and accessible only to authorised project team members.

Second, the research team will check transcripts during the transcription process for statements that may threaten participant anonymity (e.g., mentioning others’ full names, specific institutional characteristics, or identifiable events), and these will be appropriately revised or deleted after discussion by the research team.

Third, results will be presented with anonymised quotations in publications. Quotations will use codes indicating participant category (e.g., “A01-FP” for the first participant (general practitioner), “B02-M” for the second participant (manager)) without disclosing any other identifiable information.

Fourth, the informed consent process will clearly state that participation in this study is entirely voluntary, that participants may withdraw at any time, and that withdrawal will not have any adverse effect on their job evaluation, performance assessment, or relationship with the research unit.

## Data management

Study data will be retained for 5 years after study completion, stored on the university's encrypted institutional servers and in restricted-access project folders to which only authorised research team members have access; paper materials will be kept in locked cabinets. Data security management includes: dual backup of electronic data; regular integrity checks; tiered access control; and data sharing in compliance with relevant data protection regulations. Interview audio recordings will be deleted once transcription and verification are complete (expected within six months of each interview), and only anonymised transcripts will be retained. Anonymised quantitative data and analysis materials may be made available to bona fide researchers on reasonable request or via an open repository, subject to ethical and data-protection approvals. Any data sharing beyond the scope of the original ethical approval will require re-approval from the ethics committee.

## Patient and public involvement

Patients and the public were not involved in the initial design of this protocol. As described in the Patient and Public Involvement (PPI) Plan above, a Community Advisory Board comprising patients, family caregivers, and a community health leader will be established prior to Phase II to support questionnaire adaptation, to assist with the interpretation of preliminary qualitative findings, and to co-develop plain-language summaries for dissemination. In addition to these advisory roles, patients will participate as survey respondents in the quantitative phase. Study results will be disseminated to participating communities through policy briefs and public reports.

## Dissemination plan

Study results will be disseminated through:

Submission to international peer-reviewed journals (e.g., BMJ Open, Implementation Science);

Presentation at national and international academic conferences;

Policy briefs to the Health Commission of the Inner Mongolia Autonomous Region and participating banner/county health commissions;

Seminars and lectures at primary healthcare institutions and academic institutions.

Reports will adhere to the COREQ reporting standards (39) and the SRQR reporting standards (41) to ensure transparency and reproducibility of qualitative methods.

## Appendix. Structural equation modelling (SEM) analysis plan (prepared in accordance with JARS-SEM)

This appendix specifies the full analysis plan for the structural equation model described in the main text (Section “Structural equation modelling”), prepared in accordance with the Journal Article Reporting Standards for structural equation modelling (JARS-SEM; APA Publications and Communications Board Working Group, 2008) so that the model can be independently evaluated and replicated.

Model specification. Measurement model. Each latent construct will be measured by multiple indicators from the instruments described in the main text and Table 6: awareness, trust in the primary healthcare system, perceived usefulness, perceived ease of use, privacy concerns, perceived risk, and social influence, together with the three COM-B mediators (capability, opportunity, and motivation) and the outcome variable (patient acceptance of AI-assisted diagnosis). Each latent variable will be scaled by fixing one factor loading to 1.0, and the uniquenesses of all indicators will be freely estimated. Cross-loadings will be constrained to zero unless theoretically justified and explicitly reported.

Model specification. Structural model. The structural model specifies (a) direct paths (H1) from the patient-level contextual variables to patient acceptance of AI-assisted diagnosis, and (b) indirect paths through the three COM-B mediators (H2–H4). All paths will be estimated simultaneously. Model identification will be verified using the t-rule and, where necessary, the two-indicator rule; the final measurement model will be confirmed by confirmatory factor analysis (CFA) before the structural paths are added.

Estimation. Parameters will be estimated by maximum likelihood. Because the data are expected to deviate from multivariate normality, the Satorra–Bentler robust estimator (MLR) will be used for fit statistics and standard errors. Indirect effects will be tested using bias-corrected bootstrap confidence intervals based on 5,000 resamples.

Missing data and non-normality. As described in the main text, questionnaires with more than 20% missing responses on core scales, or with obvious patterned responding, will be excluded. For remaining item-level missing data, full-information maximum likelihood (FIML) estimation will be used within the SEM framework (Mplus), and multiple imputation will be applied for the regression analyses if the proportion of missingness exceeds 5%. Variables with marked non-normality will be examined, and the Satorra–Bentler (MLR) robust estimator will be used where needed.

Sample size and power. As described in the Sample Size Calculation section, the target of a minimum of 482 valid responses exceeds the minimum required by the cross-sectional formula (382) after allowing for approximately 20% invalid or incomplete questionnaires. An *a priori* Monte Carlo simulation-based power analysis will additionally be conducted in Mplus using the final measurement model and expected effect sizes (standardised path coefficients of approximately 0.2) to confirm adequate power (≥ 0.80) for the structural paths of primary interest.

Model fit evaluation. Overall fit will be evaluated with the chi-square statistic and a set of global fit indices: CFI ≥ 0.95, TLI ≥ 0.95, RMSEA ≤ 0.06 (with 90% confidence interval), and SRMR ≤ 0.08. Nested models will be compared using the Satorra–Bentler scaled chi-square difference test and ΔCFI (≤ 0.01).

Measurement invariance and multigroup analysis. Before subgroup comparisons, configural, metric, and scalar invariance will be tested across the groups of interest (e.g., age groups, geographic area types, education levels). Invariance will be judged by ΔCFI ≤ 0.01 and ΔRMSEA ≤ 0.015. Meaningful group comparisons of path coefficients will be undertaken only where scalar (or at least partial metric) invariance is established.

Model modification. *Post hoc* modifications will be permitted only when (a) they are theoretically justifiable, (b) they are supported by modification indices of meaningful magnitude, and (c) they are documented and, where possible, cross-validated on a random split-half subsample. All modifications will be reported transparently in the results manuscript.

Reporting. In accordance with JARS-SEM, the results will report: the model specification and path diagram; the data and sample description; the software and estimator used; the correlation/covariance matrix; global and local fit indices with their criteria; standardised and unstandardised parameter estimates with standard errors and confidence intervals; bootstrap results for indirect effects; measurement invariance test results; and sensitivity analyses. Any deviation from this plan will be reported and justified.

## References

[1] World Health Organization. All for Health, Health for All: Optimizing service delivery, the driving force for primary health care and universal health coverage. Geneva: WHO (2025). Available online at: https://www.who.int/ (cited July 3, 2026).

[2] United Nations. Sustainable Development Goal 3: Ensure healthy lives and promote well-being for all at all ages. New York: UN. Available online at: https://sdgs.un.org/ (Accessed July 3, 2026).

[3] World Health Organization, International Bank for Reconstruction and Development/The World Bank. Tracking Universal Health Coverage: 2023 Global Monitoring Report. Geneva: WHO (2023). Available online at: https://www.who.int/ (cited July 3, 2026).

[4] Donabedian A. Evaluating the quality of medical care. Milbank Mem Fund Q. (1966) 44(3):166–206. 10.2307/3348969 5338568

[5] Odhus CO Kapanga RR Oele E. Barriers to and enablers of quality improvement in primary health care in low- and middle-income countries: a systematic review. PLOS Glob Public Health. (2024) 4(1):e0002756. 10.1371/journal.pgph.0002756 38236832 PMC10796071

[6] Hyuha G Kilonzi M Sirili N Nyamle N Kiwango G Mfinanga JA. Access to emergency care in primary healthcare system in Tanzania: a mixed-method community-based study in a resource-limited setting. BMJ Public Health. (2025) 3:e003812. 10.1136/bmjph-2025-003812 41278252 PMC12636875

[7] Ugwu HC Obodo OR Okafor CN Ojukwu G Ekarika E Ejiyooye TF. Implementation of AI-driven diagnostic tools to improve access and efficiency in rural healthcare: an umbrella review. Cureus. (2026) 18(1):e101326. 10.7759/cureus.101326 41685033 PMC12892150

[8] Nesengani T Downing C Van Der Merwe L Ten Ham-Baloyi W. Barriers to effective patient care as experienced by nurses in primary healthcare clinics in African countries: a systematic review of qualitative studies. BMC Nurs. (2025) 24(1):232. 10.1186/s12912-025-02877-5 40022067 PMC11869662

[9] Means AR Kemp CG Gwayi-Chore M-C Gimbel S Soi C Sherr K. Evaluating and optimizing the consolidated framework for implementation research (CFIR) for use in low- and middle-income countries: a systematic review. Implement Sci. (2020) 15(1):17. 10.1186/s13012-020-0977-0 32164692 PMC7069199

[10] Institute of Medicine (US) Committee on Quality of Health Care in America. Crossing the Quality Chasm: A New Health System for the 21st Century. Washington (DC): National Academies Press (US) (2001). 10.17226/10027 25057539

[11] Huang M Rozelle S Cao Y Wang J Zhang Z Duan Z. Primary care quality and provider disparities in China: a standardized-patient-based study. Lancet Reg Health West Pac. (2024) 50:101161. 10.1016/j.lanwpc.2024.101161 39253593 PMC11381900

[12] Lian L Chen JY Wang XX Li YH Zhu Y. Current situation and countermeasure of medical service capacity of primary care physicians in China. Chin Gen Pract. (2023) 26(34):4246–53. 10.12114/j.issn.1007-9572.2023.0289

[13] Liu L Chen Y Xiang H Zhong J Zhou P Xiao Y. Measuring and optimizing the spatial accessibility of primary health care in remote and rural areas: a case study of Liannan Yao Autonomous County in China. BMC Health Serv Res. (2024) 24(1):1100. 10.1186/s12913-024-11556-1 39300487 PMC11414241

[14] Li M Zhang X Tang H Zheng H Long R Cheng X. Quality of primary health care for chronic diseases in low-resource settings: evidence from a comprehensive study in rural China. PLoS One. (2024) 19(7):e0304294. 10.1371/journal.pone.0304294 39052549 PMC11271947

[15] World Bank. World Bank Country and Lending Groups. Washington, D.C.: World Bank Group (2026). Available online at: https://datahelpdesk.worldbank.org/knowledgebase/articles/906519-world-bank-country-and-lending-groups (Accessed July 17, 2026).

[16] Brey Z Gitau S Miles M Bitton A. Equity by design in artificial intelligence for primary care in low-income and middle-income countries. Lancet Prim Care. (2026) 2(3):100135. 10.1016/j.lanprc.2026.100135

[17] Kabir R Williams M Rayhan N. Cognitive AI-Assisted Primary Care Health Delivery: A Pilot Study in Bangladesh. medRxiv (2026). 10.64898/2026.04.03.26349253

[18] Zhang J Li D Wang X Wu Z Lyu Q. Application and challenges of DeepSeek in primary care in China. Front Public Health. (2025) 13:1721308. 10.3389/fpubh.2025.1721308 41393030 PMC12698542

[19] Agweyu A Mateen BA Denniston AK Riley RD Menon V Korom R. Generative AI-enabled clinical decision support system in primary care: a pragmatic, cluster-randomized trial. Nat Med. (2026) 32:3032–3039. 10.1038/s41591-026-04503-6 42362867 PMC13472850

[20] Khattak M Bowness JS Yonis R Kierkegaard P McGregor A Perry DC. Navigating the barriers and facilitators to implementation of AI in healthcare: a scoping review. Bone Joint J. (2025) 107-B(7):666–72. 10.1302/0301-620X.107B7.BJJ-2024-1570.R1 40588252

[21] Li X Liu J. Artificial intelligence empowers primary healthcare services: progress and challenges. Chin Gen Pract. (2026) 29(4):436–43. 10.12114/j.issn.1007-9572.2025.0314

[22] Dantas C Cabrita M Bobowicz M Op Den Akker H Rafael-Palou X Hakkarainen T. Integrating participatory social innovation into requirements engineering for AI health care solutions: case study. J Med Internet Res. (2026) 28:e87984. 10.2196/87984 42397946 PMC13331246

[23] Agur Cohen D Heymann AD Levkovich I. Partners in practice: primary care physicians define the role of artificial intelligence. Healthcare (Basel). (2025) 13(16):1972. 10.3390/healthcare13161972 40868588 PMC12386139

[24] Dantas C Reigada C Cabrita M Mitsis A Ghiță C Geanta M. Co-research in the development of AI and digital health tools for cancer management and care: a systematic review. BMC Health Serv Res. (2026) 26:844. 10.1186/s12913-026-14620-0 42057000 PMC13277194

[25] Francisco KM Burns CM. An approach to potentially increasing adoption of an artificial intelligence-enabled electronic medical record encounter in Canadian primary care: protocol for a user-centered design. JMIR Res Protoc. (2024) 13:e54365. 10.2196/54365 39024011 PMC11294781

[26] Spurway C Flanagan S Cooper J Crowe FL Haroon S Marshall T. Patient perceptions of artificial intelligence-supported shared decision-making in UK primary care for multiple long-term conditions: qualitative study. J Med Internet Res. (2026) 28:e92518. 10.2196/92518 42398057 PMC13331396

[27] Darcel K Upshaw T Craig-Neil A Macklin J Steele Gray C Chan TCY. Implementing artificial intelligence in Canadian primary care: barriers and strategies identified through a national deliberative dialogue. PLoS One. (2023) 18(2):e0281733. 10.1371/journal.pone.0281733 36848339 PMC9970060

[28] Damschroder LJ Aron DC Keith RE Kirsh SR Alexander JA Lowery JC. Fostering implementation of health services research findings into practice: a consolidated framework for advancing implementation science. Implement Sci. (2009) 4:50. 10.1186/1748-5908-4-50 19664226 PMC2736161

[29] Damschroder LJ Reardon CM Widerquist MAO Lowery J. The updated consolidated framework for implementation research based on user feedback. Implement Sci. (2022) 17(1):75. 10.1186/s13012-022-01245-0 36309746 PMC9617234

[30] Kirk MA Kelley C Yankey N Birken SA Abadie B Damschroder L. A systematic review of the use of the consolidated framework for implementation research. Implement Sci. (2015) 11(1):72. 10.1186/s13012-016-0437-z PMC4869309 27189233

[31] Michie S Van Stralen MM West R. The behaviour change wheel: a new method for characterising and designing behaviour change interventions. Implement Sci. (2011) 6:42. 10.1186/1748-5908-6-42 21513547 PMC3096582

[32] Cane J O'Connor D Michie S. Validation of the theoretical domains framework for use in behaviour change and implementation research. Implement Sci. (2012) 7:37. 10.1186/1748-5908-7-37 22530986 PMC3483008

[33] Atkins L Francis J Islam R O’Connor D Patey A Ivers N. A guide to using the theoretical domains framework of behaviour change to investigate implementation problems. Implement Sci. (2017) 12(1):77. 10.1186/s13012-017-0605-9 28637486 PMC5480145

[34] DiMaggio PJ Powell WW. The iron cage revisited: institutional isomorphism and collective rationality in organizational fields. Am Sociol Rev. (1983) 48(2):147–60. 10.2307/2095101

[35] Emerson K Nabatchi T Balogh S. An integrative framework for collaborative governance. J Public Adm Res Theory. (2012) 22(1):1–29. 10.1093/jopart/mur011

[36] Concannon TW Meissner P Grunbaum JA McElwee N Guise J-M Santa J. A new taxonomy for stakeholder engagement in patient-centered outcomes research. J Gen Intern Med. (2012) 27(8):985–91. 10.1007/s11606-012-2037-1 22528615 PMC3403141

[37] Creswell JW Plano Clark VL. Designing and Conducting Mixed Methods Research. 3rd ed Thousand Oaks, CA: SAGE Publications (2018).

[38] Inner Mongolia Autonomous Region Bureau of Statistics. Statistical Bulletin of the Inner Mongolia Autonomous Region on the 2025 National Economic and Social Development (2026). Available online at: https://www.nmg.gov.cn/tjsj/tjsj/tjgb/202604/t20260409_2886936.html (Accessed August 09, 2026).

[39] Tong A Sainsbury P Craig J. Consolidated criteria for reporting qualitative research (COREQ): a 32-item checklist for interviews and focus groups. Int J Qual Health Care. (2007) 19(6):349–57. 10.1093/intqhc/mzm042 17872937

[40] Fernandez ME Ten Hoor GA Van Lieshout S Rodriguez SA Beidas RS Parcel G. Implementation mapping: using intervention mapping to develop implementation strategies. Front Public Health. (2019) 7:158. 10.3389/fpubh.2019.00158 31275915 PMC6592155

[41] O’Brien BC Harris IB Beckman TJ Reed DA Cook DA. Standards for reporting qualitative research: a synthesis of recommendations. Acad Med. (2014) 89(9):1245–51. 10.1097/ACM.0000000000000388 24979285

